# Expansion of Human and Murine Hematopoietic Stem and Progenitor Cells *Ex Vivo* without Genetic Modification Using MYC and Bcl-2 Fusion Proteins

**DOI:** 10.1371/journal.pone.0105525

**Published:** 2014-08-29

**Authors:** Gregory A. Bird, Avital Polsky, Patricia Estes, Teri Hanlon, Haley Hamilton, John J. Morton, Jonathan Gutman, Antonio Jimeno, Brian C. Turner, Yosef Refaeli

**Affiliations:** 1 Taiga Biotechnologies, Inc., Aurora, Colorado, United States of America; 2 Charles C. Gates Center for Regenerative Medicine and Stem Cell Biology and Department of Dermatology, University of Colorado School of Medicine, Aurora, Colorado, United States of America; 3 University of Colorado School of Medicine, Department of Medicine, Division of Medical Oncology, Aurora, Colorado, United States of America; French Blood Institute, France

## Abstract

The long-term repopulating hematopoietic stem cell (HSC) population can self-renew *in vivo*, support hematopoiesis for the lifetime of the individual, and is of critical importance in the context of bone marrow stem cell transplantation. The mechanisms that regulate the expansion of HSCs *in vivo* and *in vitro* remain unclear to date. Since the current set of surface markers only allow for the identification of a population of cells that is highly enriched for HSC activity, we will refer to the population of cells we expand as Hematopoietic Stem and Progenitor cells (HSPCs). We describe here a novel approach to expand a cytokine-dependent Hematopoietic Stem and Progenitor Cell (HSPC) population *ex vivo* by culturing primary adult human or murine HSPCs with fusion proteins including the protein transduction domain of the HIV-1 transactivation protein (Tat) and either MYC or Bcl-2. HSPCs obtained from either mouse bone marrow, human cord blood, human G-CSF mobilized peripheral blood, or human bone marrow were expanded an average of 87 fold, 16.6 fold, 13.6 fold, or 10 fold, respectively. The expanded cell populations were able to give rise to different types of colonies in methylcellulose assays *in vitro*, as well as mature hematopoietic populations *in vivo* upon transplantation into irradiated mice. Importantly, for both the human and murine case, the *ex vivo* expanded cells also gave rise to a self-renewing cell population *in vivo*, following initial transplantation, that was able to support hematopoiesis upon serial transplantation. Our results show that a self-renewing cell population, capable of reconstituting the hematopoietic compartment, expanded *ex vivo* in the presence of Tat-MYC and Tat-Bcl-2 suggesting that this may be an attractive approach to expand human HSPCs *ex vivo* for clinical use.

## Introduction

Hematopoietic stem cells (HSCs) are rare cells that reside in adult bone marrow and have the potential to give rise to the entire repertoire of mature blood cells [1]. HSCs are essential for the maintenance of all blood cell compartments [2]. Stem cell transplantation is an important adjunct in therapy for hematologic malignancy, autoimmunity and immunodeficiency [3]. Therefore, understanding the molecular mechanisms that regulate HSC self-renewal, proliferation, survival, lineage commitment and differentiation should enable more effective harnessing of stem cells for therapeutic use in regenerative medicine. The therapeutic utility of HSCs has been limited by their low frequency and inability to propagate *in vitro*. Expansion of these cells is particularly key for clinical applications such as gene therapy. The function of HSCs *in vivo* is dependent on complex microenvironmental signals that determine self-renewal, lineage commitment and differentiation. Attempts to expand HSC populations have been hampered by the inability to maintain multipotency and prevent differentiation, while allowing self-renewal [4]. Previous efforts to *ex vivo* expand stem cells capable of hematopoietic cell reconstitution involve using cytokine cocktails [5]; ligands for Notch-1 [6]; Tat-fusion proteins for HoxB4 [7], NF-Ya [8], and other transcription factors [9]; as well as small molecules (PGE2) and Aryl Hydrocarbon Receptor Antagonists [10]–[11]. The nature of the expanded cells among these different approaches varies, yielding mixed results in xenochimaeric transplanted mouse studies, and in the clinic [12]. Since the current set of surface markers only allow for the identification of a population of cells that is highly enriched for HSC activity, we will refer to the population of cells we expand as HSPCs.

We have previously observed that the retroviral transduction of murine bone marrow HSPCs with viruses encoding an inducible form of MYC and Bcl-2 yielded an Acute Myeloid Leukemia-like disease that was largely composed of cells with a surface phenotype that was lin^−^/Sca-1^+^/c-Kit^+^ [13], [unpublished results]. We were able to generate cell lines that resembled the long-term repopulating stem cells based on their surface phenotype a well as their ability to reconstitute the hematopoietic compartment of Rag-1^−/−^ mice. Further, bone marrow cells from the initial cohort of reconstituted Rag-1^−/−^ mice resulted in hematopoietic reconstitution after serial transplantation into new cohorts of Rag-1^−/−^ mice [2], [unpublished results]. In order to resolve the lingering concerns of integrated retroviral sequences in the genome, we generated Tat-fusion proteins with MYC and Bcl-2. These fusion proteins support the *ex vivo* expansion of murine and human HSCs demonstrated by self-renewal and reconstitution of the hematopoietic cell lineages *in vivo*. We report here a novel approach for expanding HSPCs derived from murine bone marrow, as well as from human cord blood (CB), from G-CSF mobilized peripheral blood of adults, or from adult human bone marrow.

## Methods

### Cloning of pTAT-MYC-V5-6xHis (Amp^R^) and pTAT-Bcl2Δ-V5-6xHis (Amp^R^)

Plasmid pTAT-MYC-V5-6xHis was made by PCR amplification of a cDNA encoding human cMYC using a forward primer that contains an in frame N-terminal 9-amino-acid sequence of the TAT protein transduction domain of HIV (RKKRRQRRR), and a reverse primer that removed the stop codon. The PCR product was then cloned into pET101/D-Topo (Invitrogen) vector, which includes a C-terminal V5 epitope and 6x-histidine purification tag. pTAT-Bcl2Δ-V5-6xHis was generated using a cDNA encoding human Bcl-2 and the same method described above. The unstructured loop (A.A. #27-80) was removed from the BCL-2 coding sequence using a Quick Change site directed mutagenesis kit (Stratagene #200521-5) according to the manufacturers' protocol.

### Bacterial strain used for protein expression

BL-21 RARE cells were created by transforming BL-21 Star *E. coli* strain (Invitrogen) with pRARE (Cam), isolated from BL21 Rosetta cells (Novagen), that express tRNAs for AGG, AGA, AUA, CUA, CCC, and GGA codons.

### Purification methods for recombinant Tat-fusion proteins

pTAT-MYC-V5-6xHis was transformed into BL21 RARE cells and grown on a TB/Amp/Cam plate at 37°C overnight. An isolated colony was used to inoculate a 100 ml TB/Amp/Cam starter culture that was grown at 37°C overnight. TB/Amp/Cam broth (1 liter) was inoculated with enough starter culture to establish an OD_600_ of 0.1 and grown to an OD_600_ of 0.5. The culture was induced with 0.5 mM IPTG at 37°C for 3 hrs. Bacteria were then pelleted by centrifugation. The cell pellet was resuspended in lysis buffer (8 M urea, 100 mM NaH2PO4, 10 mM Tris pH 7.0, 10 mM imidazole, final pH was brought to 7.2) and lysed at room temperature overnight on a shaker. The lysate was diluted in 6 M urea and brought to 450 mM NaCl, 50 mM NaH_2_PO_4_, 5 mM Tris pH 7.0. The lysate was treated with Benzonase (500 units) to remove residual nucleic acids. The lysate was cleared by centrifugation at 12,000 RPM for 60 min and filtered through a .22 µM filter. The lysate was degassed and applied to a nickel affinity column (GE) using a GE AKTA purifier 10 FPLC. The column was washed with lysis buffer containing 20 mM imidazole, followed by a gradient elution with lysis buffer containing 500 mM imidazole. The protein was refolded by dialyzing in dialysis buffer (450 mM NaCl, 50 mM NaH_2_PO_4_, 5 mM Tris pH 7.0, 5% glycerol, 1 mM DTT, final pH was brought to 7.2). Endotoxin was reduced by passing the purified protein over an Acticlean Etox colum (Sterogen). The purity and size of the proteins were verified using SDS-PAGE electrophoresis followed by either Coomassie blue or silver staining. Protein concentration was measured by Bradford protein assay (Sigma). The endotoxin level was determined using Limulus Amebocyte Lysate Pyrogent single test vial (Lonza).

pTAT-Bcl2Δ-V5-6xHis protein was induced as described above. The cell pellet was resuspended in 50 mL of lysis buffer (200 mM NaCl, 200 mM KCl, 50 mM NaH_2_PO_4_, 5 mM Tris pH 7.0, 5% glycerol, 1 mM DTT, final pH brought to 7.5) supplemented with 1 mM PMSF, 2 µg/ml Leupeptin, 0.015units/ml Aprotinin, 5 µg Hen Egg Lysozyme (HEL) per 1 L of induced protein, and immediately placed on ice for 1 hour. Then the cells were sonicated on ice (Duty cycle  = 50%, Output  = 5) for 2 sets of 2 minutes. All steps from here on were conducted at temperatures between 2°C and 8°C. The lysate was treated with 500 units Benzonase to remove residual nucleic acids. The lysate was cleared by centrifugation at 12,000 RPM for 60 min and filtered through a 0.22 µM filter. The lysate was degassed and applied to a nickel affinity column (GE) using a GE AKTA purifier 10 FPLC. The column was washed with 25 volumes of lysis buffer containing 20 mM imidazole followed by gradient elution with lysis buffer containing 500 mM imidazole. The eluted protein was dialyzed against 1 L of lysis buffer to remove the imidazole. Endotoxin was removed as described above. Concentration, purity and endotoxin levels were assessed as described above. The resulting protein is referred to throughout the manuscript as Tat-Bcl-2. pTAT-Cre-6xHis protein was induced as described above. The protein was purified as described above for Tat-MYC.

### Immunofluorescence microscopy

NIH 3T3 cells were seeded onto glass cover slips in six-well plates and grown to 30 to 40% confluence. Each well was transduced with 10 µg/ml of Tat-MYC or Tat-Bcl-2, or with no treatment as a negative control. Two hours following the protein transduction, the cells were rinsed three times with PBS and then fixed in 4% paraformaldehyde-PBS for 10 minutes at room temperature. Cells were rinsed with PBS and permeabilized in PBS supplemented with 1% bovine serum albumin (BSA) and 0.1% Triton X-100 at room temperature for 3 minutes. Cells were rinsed three times with PBS and incubated for 45 minutes with V5 mouse monoclonal antiserum (Invitrogen) diluted in PBS-1% BSA (1∶1,000). Cells were rinsed three times with PBS 1% BSA and incubated for 30 minutes with goat anti-mouse Alexa 488 secondary antibodies (Invitrogen A21121) diluted in PBS-1% BSA at (1∶25). The cells were washed two times with PBS-1% BSA, followed by two washes with PBS. Cover slips were mounted onto glass slides with a 10 µl drop of 50% glycerol with Hoechst at 1 µg/ml. Images were obtained on a Zeiss Imager Z1 Fluorescence microscope.

### Western blot

Cord blood cells were transduced with 10 µg Tat-MYC for 1 hour followed by 3 washes with PBS. Two hours post-transduction, 5×10^6^ cells were harvested and the nuclear and cytoplasmic fractions were isolated. Cells (5×10^6^) were harvested every 24 hours for the next 4 days. Nuclear and cytoplasmic protein samples from Tat-MYC transduced CB cells were prepared by lysing cells in 10 mM HEPES (pH 7.6), 10 mM NaCl_2_, 3 mM CaCl_2_, and 0.5% NP40. Nuclei were pelleted, and the cytoplasm-containing supernatant fraction was precipitated with trichloroacetic acid (TCA). Western blots were probed with anti-V5 antibody (Invitrogen), anti-human ß-actin (abcam), and goat anti-rabbit IgG-HRP or goat anti-mouse IgG-HRP (Santa Cruz Biotechnology).

### T-cell viability and proliferation assays

All mice were handled in accordance with an experimental protocol approved by the Institutional Animal Care and Users Committee at the University of Colorado School of Medicine (protocols # B-87709(03)1B-1 and 87709(09)2E. All animal procedures were performed in an AAALAC-accredited facility in accordance with the *Guide for the Care and Use of Laboratory Animals* and approved by the University of Colorado Denver Institutional Animal Care and Use Committee. The spleen was collected from a euthanized C57BL/6J mouse (Jackson Laboratory), and a single cell suspension was generated by mechanical dissociation. The cells were treated with TAC buffer (135 mM NH_4_Cl, 17 mM Tris pH 7.65) to lyse the red blood cells. T-cells were activated in C10 media (500 ml bottle RPMI 1640, 10% heat inactivated FBS (inactivated at 56°C for 45 minutes), 100 units per/ml Pen/Strep, 2 mM L-glutamine, 10 mM Hepes, MEM Non-essential Amino Acids, 0.55 mM ß-Mercaptoethanol, 1 mM Sodium Pyruvate 100 mM) supplemented with 1 µ/ml of anti-CD3 (monoclonal antibody 2C11) for two days. Live lymphoblasts were collected on a Ficoll cushion, washed in C10 media, and seeded in wells of a 24 well dish at 3×10^6^ cells per well in complete media with or without Tat fusion proteins. Viability and cell division profiles were determined by flow cytometric analysis of CFSE.

### RNA isolation

Splenic T-cells were prepared and activated as described under T-cell viability and proliferation assay. Post stimulation with anti-CD3 (48 hrs), the viable cells were collected on a Ficoll cushion, washed 3 times in C10 media, and plated at 3×10^6^ cells per well of a 24 well cluster dish. Wells were left untreated or treated with 10 µg Tat-MYC, 10 µg Tat-MYC/10 µg Tat-Bcl-2, or 10 µg Tat-Cre. After Tat-fusion protein treatment (48 hrs), 5×10^6^ cells were pelleted and then resuspended in 1 mL of RNA-Bee (Tel-Test). The RNA extraction was performed according to the manufacturer's protocol. Reverse-transcribed polymerase chain reaction was performed on half the mRNA using oligo (dT; Invitrogen) and Moloney murine leukemia virus (M-MLV) RT (Invitrogen) according to the manufacturer's protocol. The other half of the extracted mRNA was processed without reverse transcriptase. The resulting cDNA was precipitated and then resuspended in 50 µL of water.

### Quantitative PCR

Quantitative PCRs were set up with 2 µL of cDNA, 10 pmol of forward oligo, 10 pmol of reverse oligo, and SYBR GREEN PCR Master Mix (Applied Biosystems). Oligos for quantitative PCR were designed using Primer Express (Applied Biosystems): mouse GAPDH: forward CATGGCCTTCCGTGTTCCTA, reverse GCGGCACGTCAGATCCA; mouse ornithine decarboxylase (ODC): forward GCCAAAAAAACCGTGTGGAA, reverse TGTTCATTTGACTCATCTTCATCGT. Analysis was performed on an ABI 7300 (Applied Biosystems) using a standard curve and following the manufacturer's instructions.

### Cytokine preparations

Cells (293FT) were plated in 150 mm plates at 10×10^6^ cells per plate in D10 media (DMEM, 10%FBS, 100 units per ml Penn/Strep, MEM NEAA (Gibco), 2 mM L-glutamine (Gibco)). The next morning, the cells were transfected with 30 µg total DNA per plate consisting of 10 µg pcDNA3.1-SCF, 10 µg pcDNA3.1-IL-3, and 10 µg pcDNA3.1-IL-6 or 10 µg pcDNA3.1-TPO, 10 µg pcDNA3.1-Flt3-L, and 10 µg pcDNA3.1-GM-CSF using calcium phosphate transfection methods previously published [14]–[15]. The following day, the media was removed and replaced with 100 ml D10 media. Cells were incubated at 37°C/5% CO2 for 4–5 days. The media was collected, sterile filtered, and frozen at −20°C in 30 ml aliquots.

### Expansion of murine HSPCs

Cohorts of five, 4–6 week old female C57BL/6J mice were obtained from Jackson Laboratories (Bar Harbor, ME). Mice were euthanized in accordance with University protocol, as stated above, and bone marrow cells were collected from the tibia and femur bones. The bone marrow cells were washed and pelleted in a 50 ml conical tube by spinning at 1,200 RPM for 5 min. The red blood cells were lysed by incubation in 5 ml sterile TAC buffer (135 mM NH_4_Cl, 17 mM Tris pH 7.65). The remaining cells were washed twice in D10 media. The remaining cell pellet was resuspended in BM medium (DMEM containing 10% FCS, 100 units per ml Penn/Strep, MEM NEAA (Gibco), 10 mM HEPES, recombinant murine IL-3, IL-6, and SCF) supplemented with 5 µg/ml recombinant Tat-MYC, and 5 µg/ml recombinant Tat-Bcl-2. Cells were cultured for 28 days and then stained for cell surface markers and assessed by flow cytometry according to the antibody suppliers' protocols. Antibodies used for immunophenotypic characterization were specific for cell surface markers (Sca-1, c-Kit, Flk-2, CD150 (SLAM), CD48 (SLAM), Mac-1, GR-1, B220, TCRß, Ter119).

Cohorts of 10 C57BL/6J mice were treated with 5 mg/mouse of 5-fluorouracil (5FU), administered intraperitoneally. Five days after 5FU treatment, bone marrow cells were collected from the femurs and tibias of the mice. The cells were prepared using the same methods as the cells from the whole bone marrow, and seeded in wells of a 24 well tissue culture dish at a density of 1×10^6^ cells per well in 1 ml of medium. The cells were split into additional wells as cell density increased, to maintain a cell density at 1×10^6^ cells per well. All culture and immunophenotypic characterization methods were identical to the whole bone marrow methods described above.

### Preparing human cord blood mononuclear and CD34^+^ cells for expansion

Fresh cord blood cells were obtained from the University of Colorado cord blood bank http://www.clinimmune.com/cordbloodbank/. Cord blood samples did not meet Clinimmune's criteria for banking, therefore were donated to research. All mothers sign consent authorizing the use of cords for research in the event the units were not banked. All human cells were de-identified and exempt from IRB oversight. No residual identifying information was gathered from these cells at any point in time. The total volume was split into 20 ml aliquots, using 50 ml conical tubes. The 20 ml aliquots were diluted 1∶1 in phosphate buffered saline (PBS). The 20 ml of diluted cord blood cells were gently overlaid over 20 ml of Ficoll-Paque Plus (Amersham Biosciences Cat # 17-1440-03). The cells were spun at 900× gravity for 60 min with the brake off. The buffy coat was removed with a glass pipette and washed twice with PBS. The cells were resuspended in CB media consisting of Iscove's media (Gibco) supplemented with 10% human plasma, 100 units per ml Penn/Strep, 60 ml of media containing SCF, IL-3 and IL-6 and 60 ml of media containing TPO, FLT3-L, and GM-CSF described above. CB media was further supplemented with 5 µg/ml recombinant Tat-MYC, and 5 µg/ml recombinant Tat-Bcl-2. Cells were incubated in 10 ml CB media with Tat-fusion proteins for 60 min at 37°C. Cells (5×10^6^ per ml) were seeded in the G-Rex 10 cell expansion device (Wilson Wolf Manufacturing) according to the manufacturer's recommendation. Cells were stained for cell surface markers and assessed by flow cytometry according to the antibody suppliers' protocols. Antibodies used for stem cell characterization are antibodies against cell surface markers (CD45, CD34, CD38, CD45RA, CD90, c-Kit, Thy 1, CD133, CD150 (SLAM), CD48 (SLAM), CD11b, B220, CD3, CD13, CD33, CD71, and GPA).

In the cases where CD34^+^ cells were isolated, the CB cells were expanded as described above. The CD34^+^ cells were then isolated using a MACS CD34^+^ cell isolation kit according to the manufacturer's protocol (Miltenyi Biotec).

### Expansion of G-CSF mobilized adult peripheral blood mononuclear and CD34^+^ cells

G-CSF mobilized patient blood samples were collected from patients undergoing transplant at the University of Colorado. All donors signed Colorado Multiple Institution Review Board (COMIRB) approved consent authorizing use of samples for research (COMIRB Protocol 08-0552 and 06-0720). The Colorado Multiple Institution Review Board approved the use of these samples for this study. G-CSF mobilized cells were received in a 1 ml volume. All G-CSF samples were de-identified and no further identifying information was associated with the cells used for these studies. The 1 ml aliquot of elutriated peripheral blood was added drop wise to 10 ml of media containing cytokine as outlined in the human cord blood cells expansion section above. The cells were washed twice in CB media and treated with 5 µg/ml recombinant Tat-MYC and 5 µg/ml recombinant Tat-Bcl-2 in a 10 ml volume of CB media for 60 min at 37°C. Cells (5×10^6^ per ml) were seeded in the G-Rex 10 cell expansion device (Wilson Wolf Manufacturing) according to the manufacturer's recommendation. Cells were stained for cell surface markers and assessed by flow cytometry as described above.

### Expansion of human adult bone marrow mononuclear and CD34^+^ cells

Adult bone marrow was collected from patients undergoing transplant at the University of Colorado. All donors signed Colorado Multiple Institution Review Board (COMIRB) approved consent authorizing use of samples for the research (COMIRB Protocol 06-0720). The Colorado Multiple Institution Review Board approved the use of these samples for this study. Adult bone marrow cells were received in a 1 ml volume. All adult bone marrow samples were de-identified and no further identifying information was associated with the cells used for these studies. The 1 ml aliquot of elutriated adult bone marrow was washed in 10 ml of CB media and treated with 5 µg/ml recombinant Tat-MYC and 5 µg/ml recombinant Tat-Bcl-2 in a 10 ml volume for 60 min at 37°C. Cells (5×10^6^ per ml) were seeded in the G-Rex 10 cell expansion device (Wilson Wolf Manufacturing) according to the manufacturer's recommendation. Cells were stained for cell surface markers and assessed by flow cytometry as described above.

### Mouse transplantation - assessment of hematopoietic stem cell function by an *in vivo* assay after *ex vivo* expansion

The nucleated cell population expanded from cord blood was injected into NOD/SCID/gamma chain^−/−^ (NSG) mice (Jackson Laboratory) that received 1.5 Gy of radiation just prior to injection. The heterologous cell population containing, in addition to different categories of mature cells, precursors and committed progenitors, and the expanded HSCs, was washed 3 times in PBS and injected into NSG mice via the tail vein in 200 µl PBS. Irradiated NSG mice were maintained on food supplemented with Uniprim and water supplemented with 5 ml of a Trimethoprim and Sulfamethoxazole suspension per 300 ml H20 (Septra, Hi-Tech Pharmacal, Sulfamethoxazole and trimethoprim oral suspension, USP 200 mg/40 mg per 5 ml). The mice were bled via the tail vein 8 weeks post-transplant to assess reconstitution by flow cytometry. Peripheral blood cells were stained with the following antibodies: anti-human CD3 (hCD3) (Biolegend Cat # 300312), anti-human CD19 (hCD19) (Biolegend Cat # 302208) and anti-human CD45 (hCD45) (Biolegend Cat # 304028). Transplantation into Rag1^−/−^ mice (Jackson Laboratory) was carried out as described above except these mice received 3.5 Gy of radiation just prior to injection of the expanded bone marrow cells via the tail vein. Expanded human HSC were studied in the context of transplantation into NSG mice, whereas murine expanded HSC were studied in the context of transplantation into Rag-1^−/−^ mice.

### Tissue harvesting from engrafted mice

All mice were handled in accordance with an experimental protocol approved by the Institutional Animal Care and Users Committee at the University of Colorado School of Medicine (protocols # B-87709(03)1B-1 and 87709(09)2E). All animal procedures were performed in an AAALAC-accredited facility in accordance with the *Guide for the Care and Use of Laboratory Animals* and approved by the University of Colorado Denver Institutional Animal Care and Use Committee. The bone marrow, spleen and thymus were collected from euthanized mouse chimeras. The bone marrow cells were collected from the tibia and femur bones. The spleen and thymus were collected, and a single cell suspension was generated by mechanical dissociation. The cells were treated with TAC buffer (135 mM NH_4_Cl, 17 mM Tris pH 7.65) to lyse the red blood cells. Cells were stained for cell surface markers and assessed by flow cytometry according to the antibody suppliers' protocols. Antibodies used for murine cell characterization are antibodies against the cell surface markers c-Kit, Sca-1, CD48, CD150, CD11b, GR-1, B220, CD19, IgM, CD3, CD8, CD4, Flk-2 and Ter119. Antibodies used for human cell characterization are antibodies against the cell surface markers CD45, CD34, CD38, CD33, CD11b, CD19, CD71, and GPA. To characterize NSG mouse engraftment, cells were always gated on human CD45 and then assessed for the other cell surface markers shown above. For the purpose of serially transplanting the harvested bone marrow, the bone marrow cells were kept sterile and washed 3 times in PBS. The cells were counted, and 1×10^6^ cells were injected into irradiated recipient mice in 200 µl PBS as described above.

### Statistical analysis

Statistical analysis to generate error bars was performed using Microsoft Excel software. Unless otherwise stated, error bar indicate the standard deviation from 3 separate wells within the same experiment.

## Results

### Generation of biologically active Tat-MYC and Tat-Bcl-2 fusion proteins

**Figure 1:**
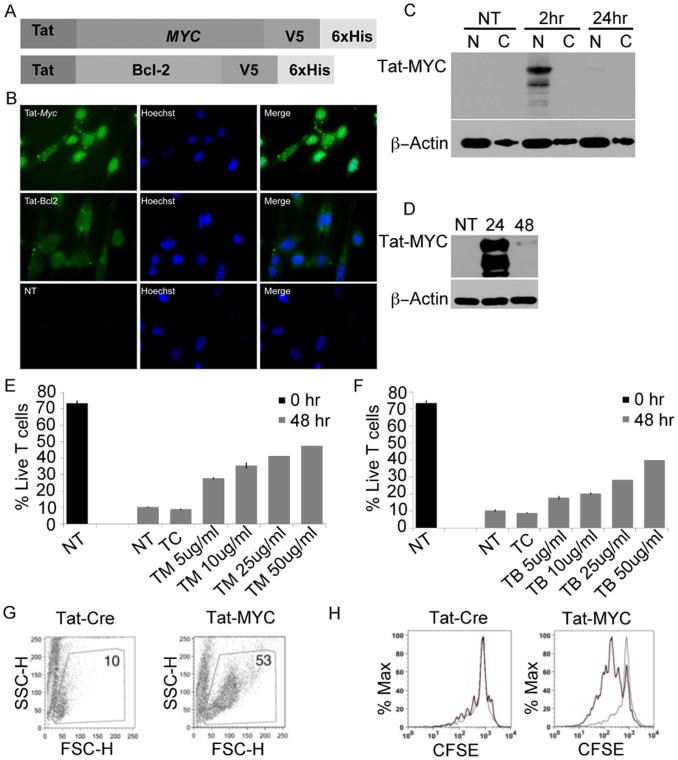
Generation and *in vitro* characterization of Tat-fusion proteins. **A.** Graphic representation of Tat-MYC and Tat-Bcl-2 fusion proteins. We cloned the cDNAs for either human MYC or human Bcl-2 in frame with the protein transduction domain of HIV-1 Tat as well as a V5 epitope tag and 6xHis tags. **B.** The fusion proteins localize to the appropriate intracellular compartment. A lawn of confluent 3T3 cells was exposed to either purified recombinant Tat-MYC or Tat-Bcl-2 or left untreated (NT). The cells were washed 2 hours later, fixed and stained with a monoclonal antibody to V5 (Green) and with a Hoechst 9934 nuclear stain (Blue). The Tat-MYC protein largely localized to the nuclear region in this timeframe, whereas the Tat-Bcl-2 remained in the cytoplasmic and perinuclear space. **C.** Tat-MYC rapidly localizes to the nucleus in primary human HSPCs. Human cord blood derived cells were pulsed with a single exposure of Tat-MYC. The cells were washed 1 hour later and the cells were collected at the indicated time points for analysis. The cells were lysed in a NP-40 buffer in order to separate the plasma membrane and cytoplasmic fraction from the nuclear fraction. All samples were analyzed by SDS-PAGE and western blot using monoclonal antibody to V5 and ß-actin. **D.** Tat-MYC was present in the nuclear fraction for a transient period of time. Human cord blood cells were treated as described for panel C. The nuclear fractions were analyzed by SDS-PAGE and western blotting using a monoclonal antibody to the V5 tag. The bulk of the protein was lost between 24 and 48 hours. There was no detectable protein left at any point after 48 hours. **E. and F.** To test the function of Tat-Myc and Tat-Bcl-2, primary murine CD4^+^ T-cells were activated with monoclonal antibody to CD3 for 48 hours. The frequency of live cells was measured in the starting population of cells (black bars). The cells were then replated in media alone (NT), or media supplemented with Tat-Cre (TC) or increasing concentrations of either Tat-MYC (TM) (**E**), or Tat-Bcl-2 (TB) (**F**). Activated T-cells cultured with Tat-MYC, but not Tat-Cre, retain a blasting phenotype and continue to proliferate after the antigenic stimulation **G.** To further test the biological function of recombinant Tat-MYC, primary murine CD4^+^ T-cells were CFSE labeled and activated *in vitro* with monoclonal antibody to CD3 for 48 hours. After activation, the cells were washed and the live cells were isolated by ficoll-hypaque centrifugation. The cells were then replated in media supplemented with 5 µg/ml Tat-Cre (first panel) or 5 µg/ml Tat-MYC (second panel). The frequency of live cells was measured by flow cytometry. **H.** The activated T-cells were further assessed by CFSE signal in order to determine whether the addition of Tat-MYC can promote proliferation in addition to survival.

We generated fusion proteins of the HIV-1 Tat protein transduction domain and either the Open Reading Frame (ORF) for human MYC, or a truncated form of human Bcl-2, that was deleted for the unstructured loop domain [16]. The recombinant proteins also encoded a V5 peptide tag as well as a 6-His tag, to enable detection and purification (Figure 1A). The proteins were synthesized in E. coli and purified to near homogeneity (Figure S1). We first tested the ability of the recombinant purified Tat-MYC and Tat-Bcl-2 to localize to the appropriate intracellular compartment. We incubated 3T3 cells with 10 µg of either Tat-MYC or Tat-Bcl-2 for 2 hours. The cells were then washed, fixed and stained with a monoclonal antibody to the V5 epitope tag. Figure 1B shows that Tat-MYC rapidly localized to the nucleus, whereas Tat-Bcl-2 remains in the cytoplasm. We also confirmed that Tat-MYC localizes to the nucleus by Western analysis of nuclear (N) and cytoplasmic (C) fractionations from cord blood cells transduced with Tat-MYC. We incubated cord blood cells with 10 µg of Tat-MYC for 1 hour and then washed the cells. Cells were collected at 2, 24, 48 and 72 hours post-transduction followed by isolation of the nuclear and cytoplasmic proteins. We observed that Tat-MYC localized to the nucleus of the cells within 2 hours post-transduction (Figure 1C, 2 hr time point, N lane). Furthermore, we observed that Tat-MYC was mostly degraded after 48 hours in the cells and no longer detectible 72 hours post-transduction (Figure 1D and data not shown).

**Figure 2:**
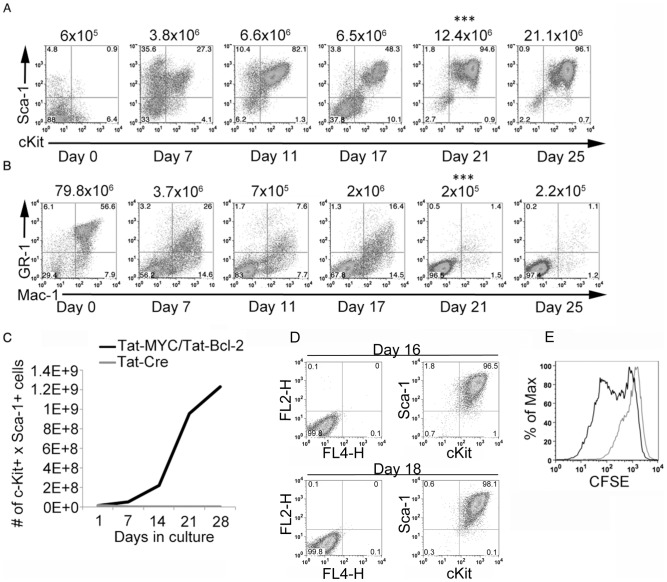
Expansion of murine HSPCs *ex vivo* with Tat-MYC and Tat-Bcl-2. We obtained the bone marrow cells from adult C57BL/6J mice. The bone marrow cells were cultured *ex vivo* for 25 days in the presence of Tat-MYC and Tat-Bcl-2 or Tat-Cre as a control protein. **A.** The c-Kit^+^/Sca-1^+^ surface phenotype of cells expanded in culture with Tat-MYC and Tat-Bcl-2 is shown. The number of days in expansion culture is shown below the flow panel. The total number c-Kit^+^/Sca-1^+^ cells is shown above the flow panel. All cultures were seeded with the heterogeneous bone marrow cell population harvested from C57BL/6J except the panel shown on day 21 with *** shown above the panel. The BM donor mice, shown on day 21, were treated with 5FU 5 days prior to the BM harvest. **B.** The cells that grew in cultures supplemented with Tat-MYC and Tat-Bcl-2 (**2A**) were negative for lineage markers Mac-1and Gr-1. **C.** Kinetics of cell expansion *ex vivo* cultures with Tat-MYC/Tat-Bcl-2. The culture of murine bone marrow cells expanded with Tat-MYC/Tat-Bcl-2 lead to a 66 fold increase in the number of c-Kit^+^/Sca-1^+^ cells in a 28 day period (black trace). By contrast, bone marrow cultured with Tat-Cre did not increase in cell number over the same time period (gray trace). **D.** CSFE labeled cKit^+^/Sca-1^+^ cells expanded in culture with Tat-MYC and Tat-Bcl-2 for 16 days (top row 2^nd^ panel) and 18 days (bottom row 2^nd^ panel) compared to the unstained control (both rows 1^st^ panel). **E.** The CFSE labeled cells from panel D revealed that cells are undergoing proliferation from day 16 (gray trace) to day 18 (black trace).

We wished to examine whether the internalized Tat-MYC retained its function by monitoring transcription of a gene whose expression is regulated by MYC, ornithine decarboxylase (ODC) [17]. T-cells from C57BL.6J mice were treated with 10 µg of Tat-MYC, 10 µg Tat-MYC/10 µg Tat-Bcl-2, or 10 µg Tat-Cre. RNA was harvested 48 hours later and used to generate cDNA. The cDNA was used in subsequent qPCR reactions. Figure S2 shows that ODC transcripts increase in cells treated with Tat-MYC or Tat-MYC and Tat-Bcl-2. We did not observe the same increase in ODC transcripts in cells treated with a control fusion protein, Tat-Cre (Figure S2).

In order to test the biological function of the recombinant purified Tat-fusion proteins we had generated, we sought to determine whether these proteins could rescue activated murine CD4^+^ T-cells from apoptosis that occurs following cytokine deprivation. We have previously used a genetic approach to show that this form of apoptosis is regulated by both MYC and Bcl-2 [18]. Figure 1E and 1F show that in a dose-dependent manner, Tat-MYC (TM) and Tat-Bcl-2 (TB), but not Tat-Cre (TC) were able to rescue activated primary CD4^+^ T-cells from cytokine-withdrawal-induced apoptosis. In addition, we have previously shown that a surfeit of MYC in lymphocytes is able to promote hyperproliferation following antigenic stimulation, using a genetic approach [18]. Figure 1G and 1H show that primary murine CD4^+^ T-cells labeled with CFSE and activated in the presence of Tat-MYC showed a robust proliferation when compared to cells activated in the presence of Tat-Cre.

### Expansion of murine bone marrow derived HSPCs with Tat-MYC and Tat-Bcl-2

We next examined whether the use of Tat-MYC and Tat-Bcl-2 would be able to support the expansion of phenotypically-defined cell populations enriched in murine stem cells *ex vivo*. We isolated the bone marrow containing the heterogeneous cell population from 5 adult C57BL/6J mice. The bone marrow cells were maintained in culture in the presence of IL-3, IL-6, SCF as well as Tat-MYC and Tat-Bcl-2. The cells were maintained in culture for 25 days. Figure 2A and Table 1 show the cell surface expression of stem cell markers c-Kit and Sca-1 for cells that were cultured for 0, 7, 11, 17, or 25 days. In this case, each day represented bone marrow harvested from an independent cohort of C57BL.6J mice. Figure 2B and Table 1 show the cell surface expression of lineage markers Mac-1, GR-1, B220, CD3, CD48, CD150, Flk-2 and Ter119. The Tat-MYC and Tat-Bcl-2 treated cells that express high levels of c-Kit and Sca-1 shown in 2A (Day 21 & Day 25 panels), are negative for lineage markers after 21 days in culture (Figure 2A and B, Day 21 & Day 25 panels, Table 1). We define the Lin-/c-Kit+/Sca-1+ cell population as LSK cells. We next tested the expanded cell populations in vitro in order to determine whether the LSK population obtained from these cultures was also enriched for HSPCs.

**Table 1:** Expansion of murine bone marrow cells *ex vivo* with Tat-MYC and Tat-Bcl-2. FACS analysis shows a continuous enrichment of the c-Kit^+^/Sca-1^+^ population (lin^−^) whereas all other cell types decrease over a 25 day period.

Day	Mac1	Gr1	Mac1xGR1	Sca1	cKit	cKitxSca1	CD48	CD150	B220	CD3	Flk2	Ter119
0	8	6	56.6	4.8	6.4	1	ND	ND	4.6	3.2	1	22.6
7	14.6	3.2	26	35.6	4	27.3	ND	ND	7	7.2	1.3	1
11	7	1.6	7.6	10.4	1.3	82	ND	ND	2.4	2.2	0.3	1
17	14.5	1.3	16.4	4	10	48	ND	ND	1	0.3	0.3	1
21	1.5	.5	1.5	2	1	94	0.2	0.6	0	0	0	0
25	1	0.2	1	1	1	95	0.1	0.5	0	0	0	0

The pyrmidine analog 5FU has been used to enrich for long-term hematopoietic reconstituting cells of the bone marrow while reducing the number of short-term repopulating stem cells and committed progenitor cells in the myeloid and lymphoid compartments that are mainly in active cell cycle [19]–[21]. We asked if HSPCs from the bone marrow of 5FU treated C57BL.6J mice expanded similarly to the bone marrow derived HSPCs of untreated mice. We obtained 5FU-enriched bone marrow of adult C57BL/6J mice. The bone marrow cells were maintained in culture in the presence of cytokines and Tat-fusion proteins as described above for whole bone marrow derived cells. As a control, bone marrow cells were cultured in media containing cytokines and Tat-Cre. Consistent with our observations for whole bone marrow derived cells, we observed an expansion of the c-Kit+/Sca-1+ population evident by day 21 in culture (Figure 2A Day 21 panel (***)). By day 21 in culture, we observed an average of 97.2% of the cell population stained positive for c-Kit/Sca-1 cell surface markers, while remaining lineage negative for 3 independent HSC expansions (Figure 2B Day 21 panel (***) and table 2).

**Table 2:** *ex vivo* expansion of the murine c-Kit^+^x Sca-1^+^ x lin^−^ cells with Tat-MYC and Tat-Bcl-2 in three independent experiments.

	Population (%)				
Expansion	c-KitxSca-1	Mac-1xGr-1	Flk2	Ter119	Fold expansion of c-KitxSca-1 population
#1	94.6	1.4	0	0.1	102
#2	96.1	1.1	0	0	93
#3	93	0.4	0	0.2	66

Fold expansion of the bone marrow harvested from 3 independent cohorts of 5-FU treated C57BL/6J mice is shown.

We observed an average 87 fold expansion of murine of LSK cells over a 21 day period in culture (Figure 2C and Table 2). By contrast, the LSK cells from 5FU treated mice failed to expand in bone marrow media supplemented Tat-Cre rather than Tat-MYC and Tat-Bcl-2 (Figure 2C). We further examined the proliferation of the c-Kit^+^/Sca-1^+^ positive population by labeling the cells with CFSE and monitoring the cell population by flow cytometry. Bone marrow cells from 5 FU treated mice were expanded for 16 days to obtain a population of cells comprised mostly of LSK cells (Figure 2D top row second panel). The LSK cells were labeled with CFSE and monitored for 2 days. Over the 2-day period, the cells retained the c-Kit+/Sca-1+ cell surface phenotype (Figure 2D bottom row second panel). The dilution of CFSE signal that occurred over a two day period demonstrated that the c-Kit+/Sca-1+ cell population actively proliferates when maintained under these culture conditions (Figure 2E).

**Figure 3:**
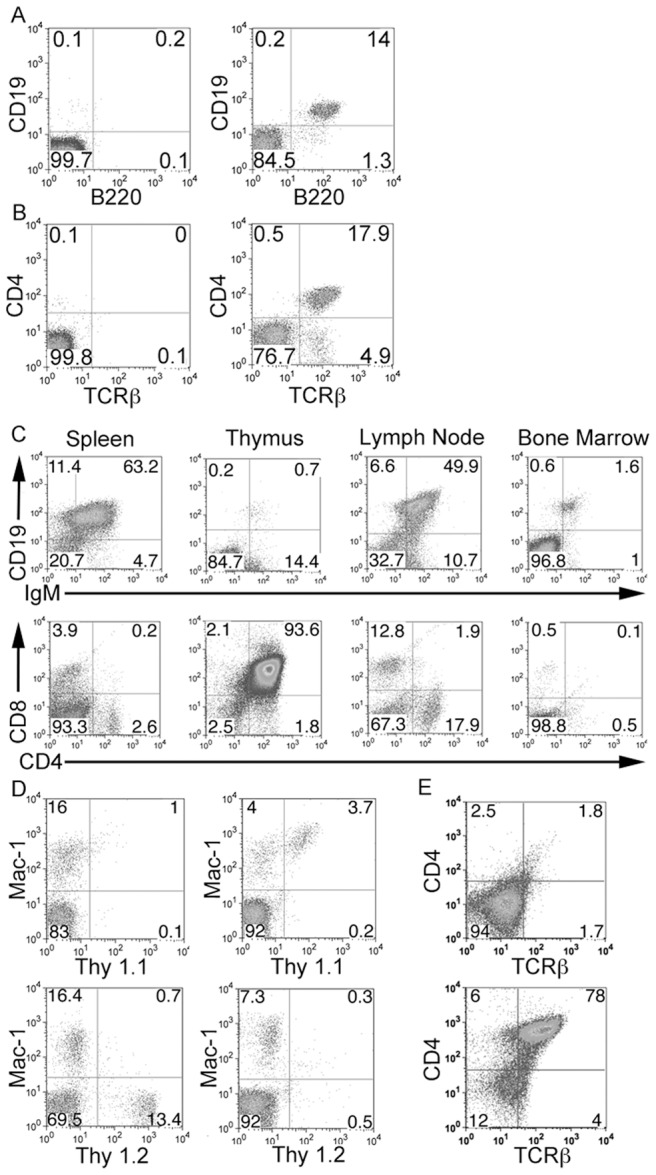
Functional analysis of expanded murine bone marrow cells *in vivo*. Cohorts of irradiated Rag-1^−/−^ mice were given 10^4^ expanded bone marrow cells obtained from 5 FU treated wild type C57BL/6J mice after expansion with Tat-MYC/Tat-Bcl-2. **A.** Peripheral blood from chimaeric mice revealed the presence of mature B-cells, expressing B-cell markers B220 and CD19, (2^nd^ panel) compared to the Rag-1^−/−^ control (1^st^ panel). **B.** Mature T-cells, expressing T-cell markers TCRß and CD4^+^, were also observed in the peripheral blood of Rag-1^−/−^ chimaeric mice (2^nd^ panel) compared to the Rag-1^−/−^ control (1^st^ panel). **C.** Lymphoid organs from the engrafted Rag-1^−/−^ mice also stained positive for developing T- (CD4 and CD8) and B-cells (CD19xIgM) by flow cytometry. **D.** The expanded mouse bone marrow also differentiates into myeloid cells. Cohorts of sublethally irradiated Rag-1^−/−^ mice (**Thy 1.2**) were given 10^4^ cells derived from bone marrow cells obtained from PLJ (**Thy 1.1**) mice after expansion with Tat-MYC/Tat-Bcl-2. Peripheral blood from the Thy 1.1/Thy 1.2 chimaeric mice revealed the presence of Thy 1.1 myeloid cells (2^nd^ panel, top row) compared to the Rag-1^−/−^ control (1^st^ panel, top row). The Thy 1.1/Thy 1.2 chimaeric mice did not stain for Thy 1.2 (2^nd^ panel, bottom row) compared to the Rag-1^−/−^ control (1^st^ panel, bottom row). **E.** 4 weeks post engraftment the Rag1^−/−^ Thy 1.1/Thy 1.2 chimaeric mice were euthanized and their thymii were assessed for T-cells. The thymus from the Rag1^−/−^ Thy 1.1/Thy 1.2 chimaeric mice stained positive for TCRßxCD4 positive T-cells (bottom panel) compared to a Rag1^−/−^ control mouse (top panel).

In order to examine the HSC content in *ex-vivo* expanded cells, we transplanted decreasing numbers of *ex vivo* expanded bone marrow cells into irradiated Rag-1^−/−^ mice. We examined the mice for the presence of mature T- and B-cells 8 weeks post-transplant. Figure 3A shows the appearance of mature B220/CD19 expressing B-cells as well as TCBß/CD4 expressing T-cells in the peripheral blood of these chimaeric mice (Figure 3B). We were able to observe the development of mature T- and B-cells in this setting following the transplantation of 10, 100 or 1000 *ex vivo* expanded bone marrow cells into irradiated Rag-1^−/−^ mice (Table 3). In addition, we also observed CD4 and CD8 positive T-cells and CD19xIgM positive B-cells in the lymph nodes, spleen, thymus and bone marrow of the chimaeric mice (Figure 3C). Mature murine T- and B-cells were obtained from the spleens of the chimaeric mice, labeled with CFSE and activated with monoclonal antibodies to CD3 (T-cells) or CD40 and IgM (B-cells). The mature lymphoid cells obtained from the chimaeric mice were able to blast and undergo cell division following activation through their antigen receptors (Figure S3). To assay the primitive, long-term repopulating stem cell activity in *ex vivo* expanded cells, we used the bone marrow cells obtained from the initial set of chimaeric mice for serial transplantation studies. Table 4 shows the frequency of mature T- and B-cells detected in the peripheral blood of irradiated Rag-1^−/−^ mice that were transplanted in a serial manner with bone marrow cells obtained from chimaeric mice generated with *ex vivo* expanded bone marrow cells. Our bone marrow derived HSCs expanded *ex vivo* were able to support hematopoietic reconstitution upon serial transplantation in 3 serial passages (Table 4).

**Table 3:** Titration of expanded bone marrow cells required for reconstitution of the lymphoid compartment in irradiated Rag-1^−/−^ mice.

	Average percent in peripheral blood	
# of BM cells transplanted	% T-cells	%B-cells
10^3^ (n = 4)	13.1	11.5
10^2^ (n = 5)	15.3	11.0
10^1^ (n = 5)	16.1	17.5
WT (n = 4)	16.5	20.3

Cohorts of Rag-1^−/−^ mice were irradiated and given transplants of either 10, 10^2^, or 10^3^ murine bone marrow cells expanded with Tat-MYC or Tat-Bcl-2. All mice were analyzed 8 weeks post-transplant.

**Table 4:** Detection of mature murine T and B-cells following serial transplantation of protein treated bone marrow cells in irradiated Rag-1^−/−^ mice.

	Average percent in peripheral blood	
Serial Transplant	% T-cells	% B-cells
1^st^ Transplant (n = 5)	8.0	14.3
2^nd^ Transplant (n = 5)	6.0	6.6
3^rd^ Transplant (n = 5)	2.7	10.4

Cohorts of Rag-1^−/−^ mice were irradiated with given a transplant of 10^3^ bone marrow cells expanded with Tat-MYC and Tat-Bcl-2. Mice were analyzed 8 weeks post-transplant for mature murine T and B-cells. Bone marrow cells were collected from the Tat-MYC/Tat-Bcl-2 treated HSPC chimaeric mice, and 10^6^ whole bone marrow cells were used for generating a second set of chimaeric mice. Likewise, this was also done a third time. FACS results were quantified and presented in this table across all of the mice in the initial transplant and two serial transplants that were carried out.

In order to demonstrate that these *ex vivo* expanded HSCs also exhibit myeloid differentiation potential, we obtained the bone marrow from 5FU treated adult PL/J mice that harbor the Thy 1.1 allele rather than the Thy 1.2 gene that is normally found in C57BL/6J. We generated chimeric mice by transplanting 10,000 *ex vivo* expanded bone marrow cells into cohorts of irradiated C57BL/6J - Rag-1^−/−^ mice. We were able to observe the development of myeloid lineage cells that were also positive for Thy 1.1, indicating that they were derived from the donor bone marrow cells (Fig 3D). In addition, we assessed these mice for the presence of mature lymphoid cells. We also observed the presence of T-cells in these chimaeric Rag-1^−/−^ mice (Figure 3E).

### Expansion of human cord blood derived HSPCs with Tat-MYC and Tat-Bcl-2

**Figure 4:**
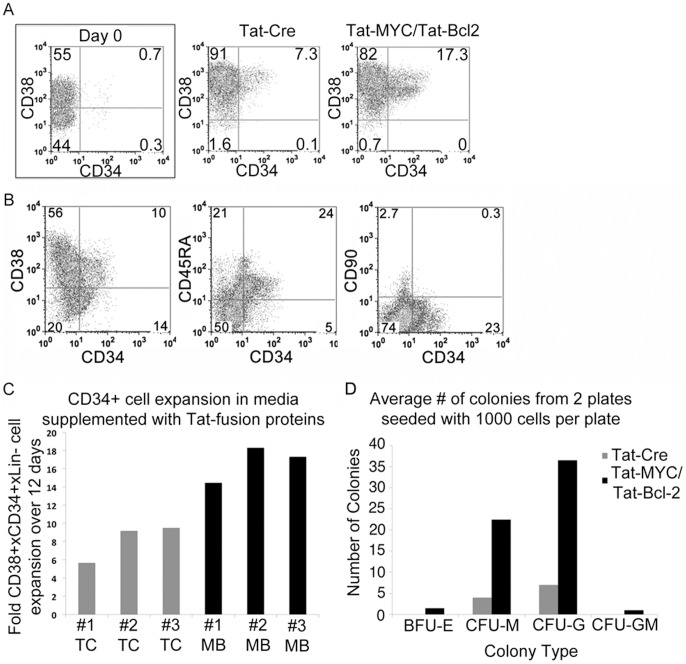
Expansion of human cord blood HSPCs with Tat-MYC/Tat-Bcl-2. Human cord blood cells were cultured *ex vivo* in CB media supplemented with Tat-Cre or Tat-MYC/Tat-Bcl-2. **A.** CD34 and CD38 cell surface phenotype of the human cord blood cells was assessed on Day 0 (1^st^ panel) or after expansion *ex vivo* for 12 days. **B.** Expanded CD34^+^ cells (1^st^ panel) were assessed for CD45RA (2^nd^ panel) and CD90 (3^rd^ panel). **C.** Graphical representation of the CD34^+^ cell expansion from three independent CB units. Each cord was split in half and treated with Tat-Cre (gray bars) or Tat-MYC/Tat-Bcl-2 (black bars). **D.** Quantification of each colony type that was observed in methylcellulose cultures seeded with 10^3^ cord blood cells cultured in CB media supplemented with Tat-Cre (gray bar) Tat-MYC/Tat-Bcl-2 (black bar).

We sought to extend our findings with the *ex vivo* expansion of murine HSPCs to human cells. We used a cytokine cocktail that was partially adapted from previously published work [5], but contained additional cytokines (IL-3, IL-6, TPO, Flt3-L, SCF, and GM-CSF). We also added recombinant purified Tat-MYC and Tat-Bcl-2 to the cocktail, or Tat-Cre as a negative control protein. We evaluated the surface phenotype of the *ex vivo* expanded human cells and observed a cell population that express CD34, CD38 and CD45 cell surface markers after extended culture in the presence of Tat-MYC/Tat-Bcl-2 (Figure 4A). The CD34^+^ cell population that expanded in the presence of Tat-MYC/Tat-Bcl-2 was also examined for expression of cells surface markers CD45RA and CD90. The CD34^+^ cells were CD45RA^lo^ and remained negative for CD90 (Figure 4B). For 3 independent cord blood units, this set of conditions resulted in an average of 16.6 fold increase in the number of CD34^+^ cells after 12 days of culture (Figure 4C black bars). We also observed CD34^+^ cell expansion in cultures expanded in the presence of Tat-Cre. However, these expansions were routinely less robust than the cultures treated with Tat-MYC and Tat-Bcl-2 resulting in an average CD34^+^ expansion of 8.1 fold for the same 3 independent cords described above with Tat-MYC/Tat-Bcl-2 treatment (Figure 4C gray bars). Similar levels of expansion were observed in experiments where the cord blood cells were cultured in a cocktail of cytokines without any added Tat-fusion proteins.

We used two different approaches to test the biological function of the expanded CD34^+^ cell population. First, we tested the content of hematopoietic committed progenitors on the basis of their ability to give rise to specific lineages *in vitro* upon plating on methycellulose media supplemented with a defined set of cytokines. Second, we tested the content of HSC by their ability to engraft following transplantation into NOD/SCID/gamma chain^−/−^ (NSG) mice and to give rise to terminally differentiated human hematopoietic cells. Figure 4D shows inside human CD34^+^ cells expanded *ex vivo* after treatment with Tat-MYC and Tat-Bcl-2 the presence of committed progenitors (BFU-E, CFU-M, CFU-G and CFU-GM) which are able to give rise of colonies *in vitro*. In addition, while the surface phenotype of the cells expanded in the presence of Tat-Cre resembled the cells expanded with Tat-MYC/Tat-Bcl-2 (Figure 4A), their BFU-E, CFU-M, CFU-G and CFU-GM colony forming unit content was significantly lower (Figure 4D).

**Figure 5:**
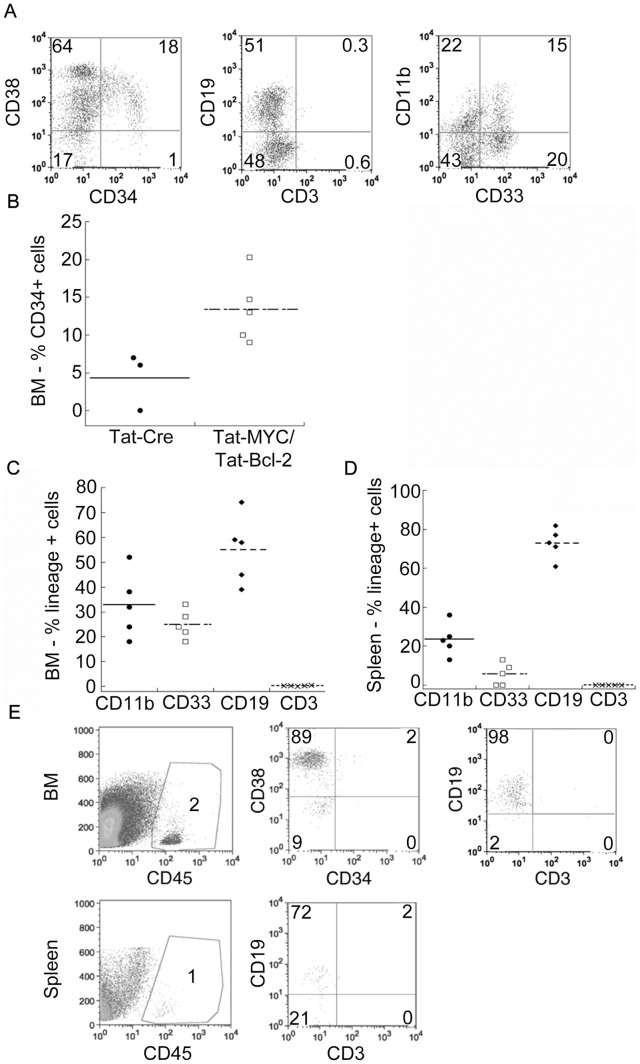
In vivo differentiation of human cord blood HSPCs expanded with Tat-MYC/Tat-Bcl-2. **A.** Cohorts of irradiated NSG mice were given transplants of 10^6^ cord blood cells expanded *ex vivo* in CB media supplemented with Tat-Cre or Tat-MYC/Tat-Bcl-2. The NSG xenochimaeric mice generated with human HSPCs cultured with Tat-MYC/Tat-Bcl-2 were euthanized and used to collect bone marrow cells. The BM cells were stained for human hCD45. Gating on CD45^+^ cells, we observed human CD34^+^/CD38^+^ cells (1^st^ panel), CD19^+^ cells (2^nd^ panel) and CD11b^+^/CD33^+^ cells (3^rd^ panel) in the bone marrow. **B.** A dot plot representation of the percent CD34^+^ engraftment in the BM of mice transplanted of 10^6^ cord blood cells expanded *ex vivo* in CB media supplemented with Tat-Cre or Tat-MYC/Tat-Bcl-2. **C, D.** A dot plot showing the lineage positive engraftment in the BM and spleen respectively of mice transplanted of 10^6^ cord blood cells expanded *ex vivo* in CB media supplemented Tat-MYC/Tat-Bcl-2. **E.** The human CD34^+^/CD38^+^ cells obtained from the bone marrow of NSG xenochimaeric mice engrafted with HSCPs expanded in CB media supplemented with Tat-MYC/Tat-Bcl-2 could be serially transplanted into a new set of NSG mice. After serial passage, 2% of the BM cells from the NSG xenochimaeric mouse stained positive for hCD45 (1^st^ panel). The hCD45^+^ cells stained positive for CD34^+^/CD38^+^ (2^nd^ panel). CD19^+^ cells were also detected in the BM (3^rd^ panel, top row) and spleen (2^nd^ panel, bottom row) of serially transplanted NSG xenochimaeric mouse.

We next tested the presence of stem cells inside human CD34^+^ cell population expanded *ex vivo* on the basis of their ability to give rise to mature human hematopoietic lineages *in vivo*. We used NSG mice as recipients for these experiments, as previously reported [22]. We transplanted of 1×10^6^ cells expanded in culture with Tat-MYC and Tat-Bcl-2 for 12 days into irradiated NSG mice. As a control, we transplanted an equal number of CD34^+^ cells expanded in culture with Tat-Cre. We euthanized the mice 10 weeks post-transplant and assessed their bone marrow for human cells. We observed human CD45^+^ cells in the bone marrow of all the xenochimaeric NSG mice that received human HSPCs expanded with Tat-MYC and Tat-Bcl-2 (Table 5). By contrast, only 2 of the 3 remaining NSG mice that received human cord blood cells expanded with Tat-Cre stained positive for human CD45 in the bone marrow. Two of the 5 mice in this cohort that received Tat-Cre treated cells did not survive to week 10. We gated on the human CD45^+^ cells and assessed the human cells for cell surface expression of CD38 and CD34 (Figure 5A). By 10 weeks post-engraftment, we observed human CD34^+^ cells in the bone marrow of 100% of the xenochimaeric NSG mice that received Tat-MYC and Tat-Bcl-2 treated human HSPC (Figure 5B, Table 5). At 10 weeks post-engraftment, we could detect human CD34^+^ cells in 66% xenochimaeric NSG that received Tat-Cre treated cells. We also observed that the number of human CD34^+^ cells in the bone marrow of NSG mice that received Tat-Cre treated cells was about 30% of the number of CD34^+^ cells in NSG mice that received Tat-MYC and Tat-Bcl-2 treated cord blood cells (Figure 5B).

**Table 5:** Detection of human CD45^+^/CD38^+^/CD34^+^ cells following serial transplantation.

Cord Blood			G-CSF mobilized patient blood		
Cord #	1^st^ engraftment	2^nd^ engraftment	Sample #	1^st^ engraftment	2^nd^ engraftment
#1	5/5	2/3	#1	3/3	2/2
#2	5/5	4/4	#2	5/5	2/4
#3	5/5	2/4	#3	5/5	2/5
#4	5/5	2/5	#4	3/5	0/5

Cohorts of NSG mice were irradiated with given a transplant of cord blood cells or G-CSF mobilized patient blood cells after a 12 day expansion with Tat-MYC/Tat-Bcl-2. The number of engrafted mice in each cohort is shown under 1^st^ engraftment. Cohorts of NSG mice were irradiated with given a transplant of 10^6^ bone marrow cells from primary xenochimaeric NSG mice. Mice were analyzed 8 weeks post-transplant for human CD45^+^/CD38^+^/CD34^+^ cells in their bone marrow compartment. The number of engrafted mice after serial transplant is shown under 2^nd^ engraftment.

We sought to further characterize the types of human CD45^+^ cells that could be found in xenochimaeric NSG mice that were generated with expanded cord blood cells. We observed human CD19 positive lymphoid cells and human CD33xCD11b positive myeloid cells in the bone marrow (Figure 5A 2^nd^ and 3^rd^ panels, and 5C) and spleen (Figure 5D) of the mice transplanted with Tat-MYC and Tat-Bcl-2 treated cells.

We next sought to determine whether the mature human B-cells we found in NSG xenochimaeric mice were responsive to stimulation of their antigen receptor. Splenocytes containing human CD45^+^/CD19^+^ human cells were labeled with CFSE and stimulated with antibodies to IgM and CD40. The cells were analyzed at 72 hours post-stimulation by flow cytometry for dilution of CFSE. Figure S4 shows the proliferation profile of the human B-cells that developed *in vivo* in xenochimaeric NSG mice generated with expanded HSPCs. This is a similar finding to what we observed previously in murine B-cells that developed in Rag-1^−/−^ mice that had received transplants of wild type murine bone marrow cells expanded *ex vivo* with Tat-MYC and Tat-Bcl-2.

Next we tested the hypothesis that within the *ex vivo* expanded cell population (generated with Tat-MYC and Tat-Bcl-2) the primitive stem cells exhibiting a long-term reconstituting ability and capable to self-renew were maintained. We used a serial transplantation approach to test this notion. We used the bone marrow cells obtained from the initial cohort of xenochimaeric NSG mice for serial transplantation studies. We transplanted 1×10^6^ unfractionated bone marrow cells harvested from the human HSPC xenochimaeric mice into a second cohort of irradiated NSG mice. The secondary cohort of xenotransplant recipient mice were euthanized 12 weeks post-transplant, and their bone marrow cells were assessed for the presence of human CD34^+^ cells by flow cytometry. We observed human CD45^+^/CD38^+^/CD34^+^ cells in the bone marrow of the secondary cohort of xenochimaeric NSG mice (Figure 5E top row, Table 5). In addition, we observed human CD19^+^ B-cells in the bone marrow and spleen of these mice (Figure 5E top and bottom row respectively).

### Comparison of *in vivo* differentiation potential in xenotransplant models between expanded cord blood derived HSPCs and unmanipulated fresh cord blood

**Figure 6:**
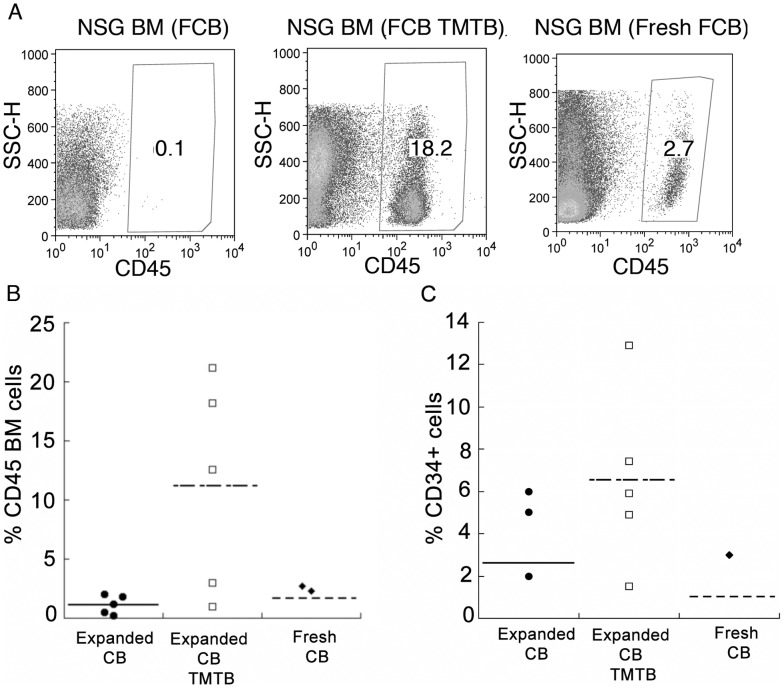
Comparison of NSG mouse engraftment with human cord blood cells expanded with Tat-MYC/Tat-Bcl-2 or fresh cord blood. **A.** hCD45^+^ cell staining in the BM of sublethally irradiated NSG mice were given transplants of 10^6^ cord blood cells expanded *ex vivo* in a cocktail of cytokines (first panel), expanded in a cocktail of cytokines supplemented with Tat-MYC/Tat-Bcl-2 (second panel), or 1×10^7^ fresh unmanipulated cord blood cells (third panel). **B.** A dot plot showing BM cells from a cohort of NSG xenochimaeric mice transplanted of 10^6^ cord blood cells expanded *ex vivo* in a cocktail of cytokines (black circles), expanded in a cocktail of cytokines supplemented with Tat-MYC/Tat-Bcl-2 (open squares), or 1×10^7^ fresh unmanipulated cord blood cells (black diamonds) were stained for human CD45. **C.** A dot plot shown human CD34^+^/CD38^+^ cells in the bone marrow 3/5 mice transplanted with cord blood expanded in cytokines alone (black circles), CD34^+^/CD38^+^ cells in the bone marrow of 5/5 mice transplanted with cord blood expanded in cytokines plus Tat-MYC/Tat-Bcl-2 (open squares), and CD34^+^/CD38^+^ cells in the bone marrow of 1 out of 3 mice transplanted with unmanipulated cord blood (black diamonds).

Next we compared cells expanded *ex vivo* in the presence of Tat-MYC/Tat-Bcl-2 to fresh cord blood. In this case, cord blood was expanded in culture for 12 days in CB media supplemented with Tat-MYC/Tat-Bcl-2 or cytokines alone. After 12 days of expansion, 1×10∧6 cells were transplanted into irradiated NSG mice. We also transplanted 1x10∧7 fresh cord blood cells, which contained a comparable number of CD34^+^ cells as the expanded cord blood, into a cohort of irradiated NSG mice. We euthanized the mice 8 weeks post-transplant and assessed the bone marrow for human cells. Figure 6A shows human CD45^+^ staining of BM harvested from a representative mouse that receive cells expanded in cytokines alone (Figure 6A, 1^st^ panel), cytokines supplemented with Tat-MYC/Tat-Bcl-2 (Figure 6A, 2^nd^ panel), or fresh cord blood (Figure 6A, 3^rd^ panel). We could detect CD45^+^ cells in the bone marrow of 100% of the xenochimaeric NSG mice that received cord blood cells expanded Tat-MYC/Tat-Bcl-2 (Figure 6B, open squares). We observed CD45^+^ cells in only 40% of the mice engrafted with fresh cord blood (6B, black diamonds). Cord blood cells expanded in cytokines alone were able to engraft 100% of the mice (6B, black circles). However, consistent with our previous observation (Figure 5), cord blood expanded in cytokines supplemented with Tat-MYC/Tat-Bcl-2 increased the number of CD45^+^ cells in the BM (Figure 6B). Next we gated on the human CD45^+^ cells and assessed the BM for cell surface expression of CD38 and CD34. We observed human CD34^+^ cells in the bone marrow of 100% of the xenochimaeric NSG mice that received Tat-MYC/Tat-Bcl-2 treated human cells (Figure 6C, open squares). While we could detect CD45^+^ cells in 100% of the mice receiving cells expanded with cytokines alone, we could only detect CD34^+^ cells in 60% of the mice (Figure 6B, black circles). Only 20% of the mice transplanted with fresh cord blood stained positive for CD34 in the BM.

**Figure 7:**
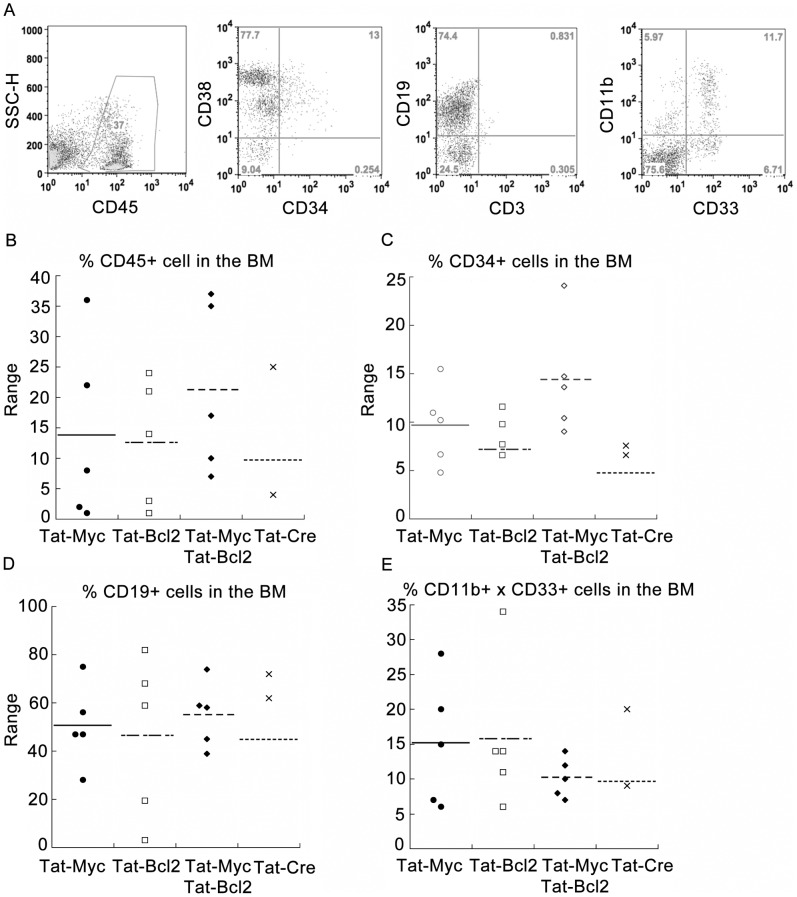
Comparison of NSG mouse engraftment with CD34^+^ cells isolated from human cord blood cells after expansion with Tat-MYC, Tat-Bcl-2, Tat-MYC/Tat-Bcl-2 or Tat-Cre. **A.** Cohorts of irradiated NSG mice were given transplants of 10^5^ CD34^+^ cells isolated from CB expanded *ex vivo* in CB media supplemented with Tat-MYC/Tat-Bcl-2. The NSG xenochimaeric mice generated with isolated CD34^+^ cells were euthanized and used to collect bone marrow cells. The BM cells were stained for human hCD45 (1^st^ panel). Gating on CD45^+^ cells, we observed human CD34^+^/CD38^+^ cells (2^nd^ panel), CD19^+^ cells (3^rd^ panel) and CD11b^+^/CD33^+^ cells (4^th^ panel) in the bone marrow. **B.** A dot plot representation of the percent CD45^+^ engraftment in the BM of mice transplanted of 10^5^ CD34^+^ cells isolated from CB expanded *ex vivo* in CB media supplemented with Tat-MYC (black circles), Tat-Bcl-2 (open squares), Tat-MYC/Tat-Bcl-2 (black diamonds) or Tat-Cre (black X). **C** A dot plot showing the percent of CD45^+^ cells shown in B that stain positive for CD34. **D and E.** A dot plot showing the lineage positive engraftment in the BM of mice shown in B.

Next we sought to determine the individual contribution of Tat-MYC and Tat-Bcl-2 to human CD34^+^ cell engraftment. We transplanted 1×10^5^ CD34^+^ cells isolated after 12 days of expansion in media supplemented with Tat-MYC, Tat-Bcl-2, Tat-MYC/Tat-Bcl-2, or Tat-Cre control protein, into irradiated NSG mice. We euthanized the mice 12 weeks post-transplant, and assessed their bone marrow for human cells. Consistent with our previous results (Figures 5 and 6), we could detect human CD45^+^ cells in the BM of mice transplanted with cord blood expanded in media supplemented with Tat-MYC/Tat-Bcl-2 (Figure 7A). We further assessed the CD45^+^ BM cells for expression of CD38/CD34, CD19, CD3, CD11b, and CD33 (all human). We could detect CD34^+^ cells, CD19^+^ B-cells and CD11b^+^/CD33^+^ myeloid cells in the BM of these mice (Figure 7A, 2^nd^, 3^rd^, and 4^th^ panel). We compared the engraftment of mice receiving CD34^+^ cells expanded with Tat-MYC/Tat-Bcl-2 to engraftment in mice receiving CD34^+^ cells expanded with Tat-MYC, Tat-Bcl-2 or Tat-Cre. Figure 7B shows human CD45^+^ cells could be detected in the bone marrow of 100% of the xenochimaeric NSG mice that received human CD34^+^ cells expanded with Tat-MYC alone, Tat-Bcl-2 alone or Tat-MYC/Tat-Bcl-2. 33% of the remaining mice that received Tat-Cre treated CD34^+^ were not engrafted. Consistent with our observations in Figure 5B, 2 of the 5 mice receiving cells expanded with Tat-Cre did not survive to week 12. We gated on the human CD45^+^ cells and assessed them for cell surface expression of CD34 and CD38. We observed human CD34^+^ cells in the bone marrow of 100% of the xenochimaeric NSG mice that received Tat-MYC alone or Tat-MYC/Tat-Bcl-2 treated CD34+ cells (Figure 7C). Only 80% of the mice receiving CD34^+^ cells expanded with Tat-Bcl-2 and 66% of the mice receiving CD34+ cells expanded with Tat-Cre had CD34^+^ cells in their BM. Interestingly, the highest levels of CD34^+^ engraftment was detected in mice transplanted with CD34^+^ cells expanded in media supplemented with Tat-MYC/Tat-Bcl-2 (Figure 7C). Next we gated on the human CD45^+^ cells and assessed the BM cells for expression of CD19, CD11b and CD33. We observed human CD19^+^ B cells and CD11b^+^/CD33^+^ myeloid cells in the bone marrow of all the xenochimaeric NSG mice (Figure 7D and E).

### Expansion of human G-CSF mobilized blood cells with Tat-MYC and Tat-Bcl-2

The use of cord blood cells for transplantation has been critical for helping to alleviate the growing demand for HSPCs. Several groups have reported methods for expanding these cells [23]. However, these same protocols fail to reliably expand G-CSF mobilized adult HSCPs. These cells are often the best source for adult transplantation, in cases where the HSPC supply is sufficient and reliable [23]. Therefore, we sought to determine whether the culture conditions we used to expand murine bone marrow cells as well as human cord blood cells would also be applicable to human adult G-CSF mobilized ones.

**Figure 8:**
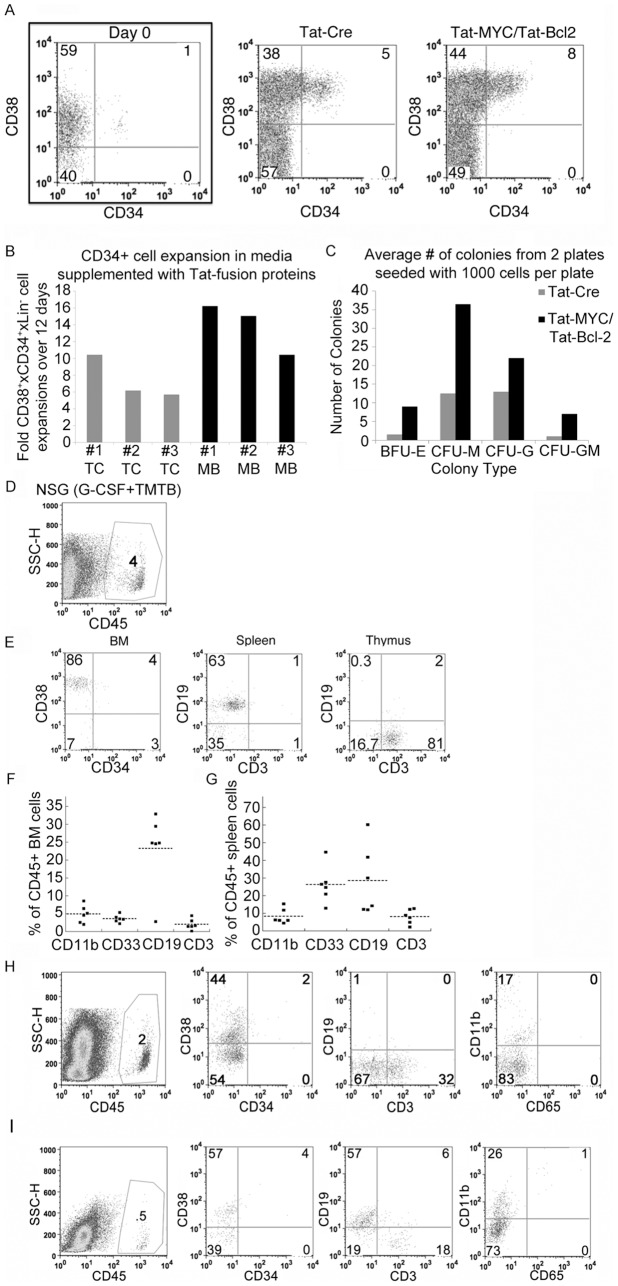
Expansion of adult human G-CSF mobilized blood cells *ex vivo* with Tat-MYC/Tat-Bcl-2. Blood cells from a G-CSF mobilized patient were cultured in CB media the presence of Tat-Cre or Tat-MYC/Tat-Bcl-2 for 12 days. **A.** The flow cytometry panels showing the percent CD34^+^ cells in culture on day 0 and day12 days of expansion in CB media supplemented with Tat-Cre or Tat-MYC/Tat-Bcl-2. **B.** Graphical representation of the CD34^+^ cells expanded from three independent G-CSF mobilized patient samples. Each sample was split in half and treated with Tat-Cre (TC) or Tat-MYC/Tat-Bcl-2 (MB). **C.** Quantification of each colony type that was observed in methylcellulose cultures seeded with 10^3^ expanded cells from G-CSF mobilized patient blood cultured in CB media supplemented with Tat-Cre (gray bar) Tat-MYC/Tat-Bcl-2 (black bar). **D.** Functional analysis of G-CSF mobilized blood-derived cells *in vivo*. Cohorts of irradiated NSG mice were given transplants of 1×10^6^ cells expanded *ex vivo* in CB media supplemented with Tat-Cre or Tat-MYC/Tat-Bcl-2. The NSG xenochimaeric mice generated with the cells expanded with Tat-Cre died between 4 and 6 weeks post transplant. 10 weeks post-transplant the NSG xenochimaeric mice generated with the human cells expanded with Tat-MYC/Tat-Bcl-2 were euthanized and used to collect bone marrow cells. The BM cells were stained for human hCD45. **E.** Gating on CD45^+^ cells, we observed human CD34^+^/CD38^+^ cells in the BM (1^st^ panel), CD19^+^ cells in the spleen (2^nd^ panel) and CD3^+^ cells in the thymus (3^rd^ panel). **F, G.** A cohort of xenochimaeric mice engrafted with 1×10^6^ G-CSF mobilized cells expanded *ex vivo* in CB media supplemented with Tat-MYC and Tat-Bcl-2 were assessed for myeloid and lymphoid cell differentiation. The CD45 positive population of bone marrow cells (**F**) and spleen cells (**G**) were analyzed for CD11b, CD33, CD3, and CD19 expression. **H.** The BM cells of NSG xenochimaeric mice engrafted with HSCPs expanded in CB media supplemented with Tat-MYC/Tat-Bcl-2 could be serially transplanted into a new set of NSG mice. After serial passage, 2% of the BM cells from the NSG xenochimaeric mouse stained positive for hCD45 (1^st^ panel). The hCD45 positive cells stained positive for CD34^+^/CD38^lo^ (2^nd^ panel). CD3^+^ cells (3^rd^ panel, top row) and CD11b^+^ cells (4^th^ panel, top row) were also detected in the BM of serially transplanted NSG xenochimaeric mouse. **I.** Mice from a cohort of xenochimaeric mice engrafted with 1×10^6^ G-CSF mobilized cells expanded *ex vivo* in CB media supplemented with Tat-MYC/Tat-Bcl-2 were assessed for long-term engraftment 12 months post-transplant. 2/5 mice had hCD45 positive cells in their BM (representative mouse shown).

We obtained 1 ml of elutriated blood each from three patients who underwent G-CSF mobilization for allogeneic stem cell transplantation. The cells were expanded *ex vivo* in CB media supplemented with cytokines as well as Tat-MYC and Tat-Bcl-2 or Tat-Cre as a control protein. Figure 8A shows the expansion of the human G-CSF mobilized CD34^+^ cells after 12 days in culture in the presence of Tat-MYC and Tat-Bcl-2 or Tat-Cre control protein. Similar to the cell surface phenotype of cord blood cells expanded for 12 days with Tat-MYC and Tat-Bcl-2 (Figure 4A), there is a distinct population of hCD45^+^/CD34^+^/CD38^+^ cells that became evident after 12 days of culture (Figure 8A). For 3 independent G-CSF mobilized blood samples, this set of conditions resulted in an average increase of 13.6 fold in the number of CD34+ cells after 12 days of culture (Figure 8B black bars). We also observed G-CSF mobilized blood derived HSCPs expanded in cultures treated with Tat-Cre. However, these expansions were routinely less robust than the cultures treated with Tat-MYC/Tat-Bcl-2 resulting in an average CD34^+^ expansion of 7.3 fold for the same 3 independent G-CSF mobilized blood samples described above with Tat-MYC/Tat-Bcl-2 treatment (Figure 8B gray bars). Our results suggest that this approach involving the use of Tat-MYC/Tat-Bcl-2 for *ex vivo* expansion of human G-CSF mobilized blood derived CD34^+^ HSPCs can generate a sufficient number of cells needed for transplantation of an average size adult according to current approaches [24].

Next, we sought to determine the content of committed progenitors in expanded cell population from human mobilized adult blood on the basis of their ability to give rise to specific lineages on methylcellulose media supplemented with a defined set of cytokines. Figure 8C shows that BFU-E, CFU-M, CFU-G and CFU-GM progenitors were able to give rise to colonies *in vitro* after *ex vivo* expansion with Tat-MYC and Tat-Bcl-2. Similar to the our observation for expanded CB cells, cells from mobilized blood, expanded with Tat-Cre, had a lower BFU-E, CFU-M CFU-G and CFU-GM colony forming unit content than the cells treated with Tat-MYC/Tat-Bcl-2 (Figure 8C).

We tested the ability of the *ex vivo* expanded human adult G-CSF mobilized cells to reconstitute irradiated NSG mice. Figure 8D shows human CD45^+^ staining of bone marrow cells obtained from a representative NSG mouse transplanted with 1×10^6^ G-CSF mobilized cells that were expanded *ex vivo* with Tat-MYC/Tat-Bcl-2. The NSG xenochimaeric mice generated with the human G-CSF mobilized cells expanded with Tat-Cre died between 4 and 6 weeks post-transplant. Analysis of lymphoid organs obtained from xenochimaeric NSG mice transplanted with G-CSF mobilized cells expanded *ex vivo* with Tat-MYC/Tat-Bcl-2 showed human CD45^+^/CD34^+^/CD38^hi^ cells in the bone marrow (Figure 8E, first panel), human CD45^+^/CD19^+^ lymphoid cells in the spleen (Figure 8E, second panel) and CD45^+^/CD3^+^ lymphoid cells in the thymus (Figure 8E, third panel).

A cohort of xenochimaeric mice engrafted with 1×10^6^ G-CSF mobilized cells expanded *ex vivo* in a cocktail of cytokines supplemented with Tat-MYC/Tat-Bcl-2 were assessed for myeloid and lymphoid cell differentiation. The human CD45^+^ population of bone marrow cells (Figure 8F) and spleen cells (Figure 8G) were analyzed for human CD11b, CD33, CD3, and CD19 expression. Both myeloid (CD11b and CD33) and lymphoid (CD3 and CD19) cell differentiation markers were observed in the bone marrow and spleen of these xenochimaeric mice.

We also assessed the ability of bone marrow cells from xenochimaeric NSG mice, generated with cells from G-CSF mobilized patient cells expanded *ex vivo* with Tat-MYC/Tat-Bcl-2, to newly engraft mice upon serial transplantation. We used the bone marrow cells obtained from the initial cohort of xenochimaeric NSG mice and transplanted of 1×10^6^ unfractionated bone marrow cells into another cohort of irradiated NSG mice. The secondary cohort of transplant recipient mice were euthanized 12 weeks post-transplant, and their bone marrow cells were assessed for the presence of human CD45^+^ cells by flow cytometry (Figure 8H, 1^st^ panel and Table 5). Similarly to what we observed with NSG mice engrafted with expanded CB cells, we observed human CD34^+^ cells in the bone marrow of the secondary cohort of xenochimaeric NSG mice upon serial transplantation (Figure 8H and Table 5). We also observed CD3^+^ T cells and CD11b^+^ myeloid cells in the BM.

We sought to determine the longevity of the hematopoietic reconstitution in xenochimaeric NSG mice, generated with cells from G-CSF mobilized patient blood expanded *ex vivo* with Tat-MYC/Tat-Bcl-2. The bone marrow cells of engrafted NSG mice were harvested 12 months post-transplant and analyzed for the presence of human CD45^+^ cells. We observed human CD45^+^ cells in 40% of the mice that received transplants of 1×10^6^ *ex vivo* expanded cells from G-CSF mobilized blood. Flow data from a representative mouse is shown in Figure 8I. We gated on the human CD45^+^ cells and assessed them for cell surface expression of CD38 and CD34. We observed human CD34^+^/CD38^lo^ cells in the bone marrow of these xenochimaeric NSG mice (Figure 8I 2^nd^ panel). Next we gated on the human CD45^+^ cells and assessed the BM cells for expression of CD19, CD3, CD11b and CD33. We observed human CD19^+^ B cells, CD3^+^ T cells, and CD11b^+^ myeloid cells in the bone marrow of all the xenochimaeric NSG mice (Figure 8I 3^rd^ and 4^th^ panel). We were able to observe mature human cells and HSPCs 12 months post-transplant in the xenochimaeric NSG mice generated with human G-CSF mobilized HSPCs expanded *ex vivo* with Tat-MYC and Tat-Bcl-2 (Figure 8I).

### Expansion of human bone marrow HSPCs with Tat-MYC and Tat-Bcl-2

**Figure 9:**
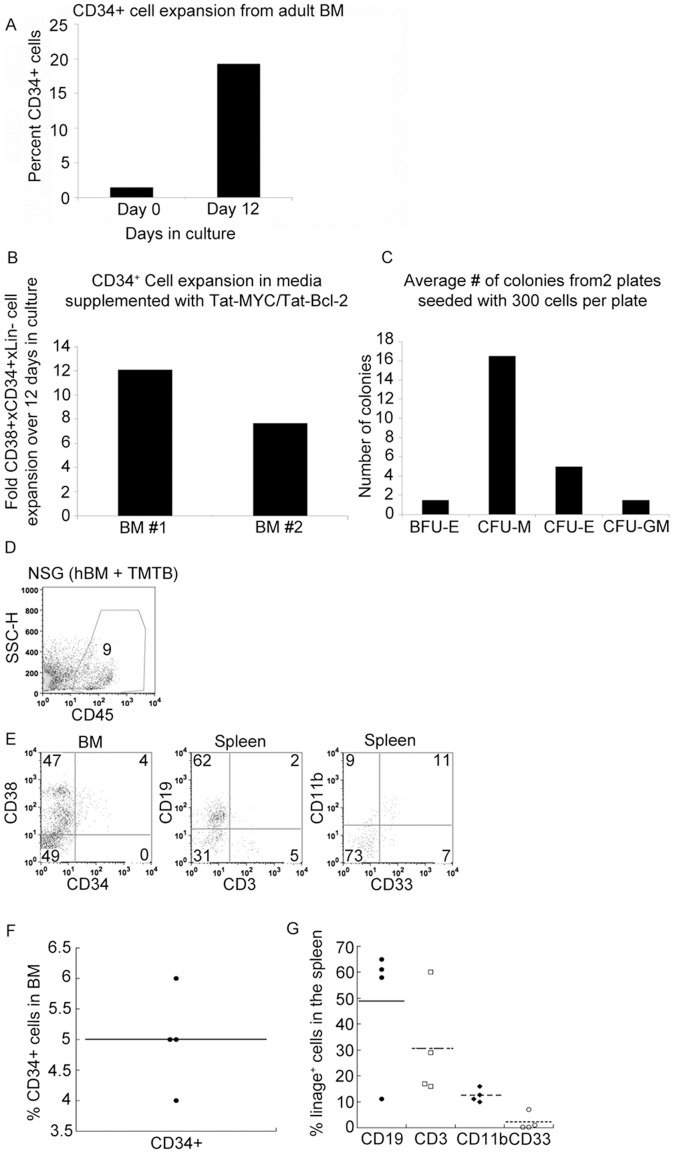
Expansion of adult human bone marrow derived cells *ex vivo* with Tat-MYC/Tat-Bcl-2. **A.** The percent CD34^+^ cells in culture on day 0 and day12 days of expansion **B.** Graphical representation of the CD34^+^ cells expansion from two independent adult BM samples. **C.** Quantification of each colony type that was observed in methylcellulose cultures seeded with 300 expanded cells from adult BM cultured in CB media supplemented with Tat-MYC/Tat-Bcl-2. Functional analysis of expanded human BM *in vivo*. Cohorts of irradiated NSG mice were given transplants of 1×10^6^ cells expanded *ex vivo* with Tat-MYC/Tat-Bcl-2. The NSG xenochimaeric mice were euthanized 10 weeks post transplant and used to collect bone marrow cells. **D.** The BM cells were stained for human hCD45. **E.** Gating on CD45^+^ cells, we observed human CD34^+^/CD38^+^ cells in the BM (1^st^ panel), CD19^+^ and CD3^+^ cells in the spleen (2^nd^ panel) and CD11b^+^/CD33^+^ cells in the spleen (3^rd^ panel). **F.** A dot plot representation of the percent CD34^+^ engraftment in the BM of mice transplanted of 10^6^ adult human BM cells expanded *ex vivo* in CB media supplemented with Tat-MYC/Tat-Bcl-2. **G.** A dot plot showing the lineage+ engraftment in the spleen of mice shown of **F**.

We sought to determine whether the culture conditions we used to expand CD34^+^ cells from human cord blood and adult G-CSF mobilized blood would work to expand CD34^+^ cells from human adult bone marrow. We obtained 1 ml of elutriated bone marrow from two patients who underwent bone marrow harvest for allogeneic transplantation. The cells were expanded *ex vivo* with media supplemented with cytokines plus Tat-MYC/Tat-Bcl-2. Figure 9A shows the CD34^+^ cell expansion of the human adult bone marrow after 12 days in culture. There is a distinct population of human CD45^+^/CD34^+^/CD38^+^ cells that became evident after 12 days of culture. We observed on average, a 10-fold expansion in the number of CD45+/CD34+/CD38+ cells derived from 2 separate adult bone marrow samples that were expanded for 12 days in CB media supplemented with Tat-MYC/Tat-Bcl-2 (Figure 9B).

Next, we sought to determine the ability of expanded committed progenitors from human adult bone marrow to give rise to specific lineages on methycellulose media supplemented with a defined set of cytokines. Figure 9C shows that adult bone marrow HSPCs expanded *ex vivo* with Tat-MYC and Tat-Bcl-2 were able to give rise to BFU-E, CFU-M, CFU-G and CFU-GM colonies *in vitro*.

In order to assay for the presence of stem cells in *ex vivo* expanded human adult bone marrow cells, we tested their ability to reconstitute irradiated NSG mice. Figure 9D shows CD45^+^ staining of bone marrow from a representative NSG mouse transplanted with 1×10^6^ cells that were expanded *ex vivo* with Tat-MYC and Tat-Bcl-2. Analysis of lymphoid organs obtained from these xenochimaeric NSG mice showed that there were human CD45^+^/CD34^+^/CD38^+^ cells in the murine bone marrow (Figure 9E first panel and 9F), as well as human CD45^+^/CD19^+^ B-cells, and CD3^+^ T-cells in the spleen (Figure 9E second panel, and 9G) and CD45^+^/CD11b^+^/CD33^+^ myeloid cells in the spleen (Figure 9E third panel and 9G) of those mice.

## Discussion

We describe here a novel approach for the expansion of murine and human HSPCs without the use of any genetic modifications. The resulting cells express cell surface markers associated with HSC and some of them retain the ability to reconstitute the hematopoietic lineages *in vivo* following irradiation, in a serial manner. A significant proportion of expanded cells belongs to the committed progenitors detected on the basis of in vitro colony-forming ability. Two key observations in these studies involve the rapid expansion of human HSPCs from multiple sources (cord blood, peripheral mobilized HSCs, and adult bone marrow) *ex vivo*, as well as the ability of the *ex vivo* expanded cells to give rise to a self-renewing cell population that is capable to establish a sustained hematopoiesis *in vivo* following transplantation.

The experiments using bone marrow from 5FU treated mice yielded a culture that was 94.6% LSK cells after 21 days in culture. These cultures were seeded on day 1 with 10×10∧6 total cells, 1×10∧6 cells harvested from each of 10 mice. Estimates of the number of long-term HSC cell population in murine bone marrow would be around 1 in 10,000 [2]. Of the 10×106 cells, we could estimate about 1000 long-term HSCs, based on previously published results [2]. Absent any expansion of the 1,000 HSC, over the 21 day expansion were the initial 10×106 cells increased to 87×10∧6 over 21 days, the HSC population would account for 1 out of every 87,000 cells. The observation that 10 cells from the expanded LSK cell cultures are capable of engrafting each Rag 1^−/−^ mouse in a cohort of 5 mice and that the bone marrow of these chimaeric mice can be serially transplanted to engraft new recipient mice suggests that the long-term HSCs are increasing in cell number over the 21 day period.

The precise functions of exogenously added *MYC* and Bcl-2 in generating this phenotype are unclear. It appears that the function of *MYC* prevents exit of HSCs from the cell cycle, driving their proliferation and inhibiting their differentiation. Signals provided by cytokines such as IL-3, IL-6, SCF and others can play a critical role in maintaining the HSC phenotype of proliferating HSC cells, when used in combinations and under the appropriate conditions. The survival function provided by Bcl-2 apparently enables rescue of HSC cells from the apoptotic death that would normally follow withdrawal of *MYC* function. This may be a critical step in enabling the HSCs to regain their ability to use physiologically available survival signals *in vivo*. Upon adoptive transfer, Bcl-2 survival function likely allows cells to habituate to microenvironmental signals provided by the bone marrow stem cell niche. In conditions of need, such as radiation-induced lymphopenia, these signals drive differentiation of the expanded HSCs to generate functional lymphoid cells and other differentiated blood cells. Importantly, we have not observed any leukemias in mice reconstituted with HSCs expanded *ex vivo* with Tat-MYC and Tat-Bcl-2 fusion proteins. This observation stands in contrast with some of the findings we had with the conditionally immortalized HSC cell lines that we had previously generated with retroviral approaches.

The approach described here to expand long-term HSCs holds several advantages over other approaches tried to date. The use of the protein transduction domain of HIV-Tat to deliver MYC and Bcl-2 protein to HSPCs removes the need for genetic modifications to express the same proteins. Tat-MYC and Tat-Bcl-2 proteins are degraded by the cells avoiding the sustained presence of MYC and/or Bcl-2 required for oncogenic transformation. However, Tat-MYC and Tat-Bcl-2 appear to be present in the cell long enough to transiently provide the signals to drive proliferation and survival, while preventing differentiation and apoptosis. Other technologies have achieved expansion of some hematopoietic precursors from cord blood, Tat-MYC and Tat-Bcl-2 are able to expand HSPCs from mouse BM, human cord blood cells, G-CSF mobilized patient blood and human BM. The use of Notch ligands to expand cord blood cells appears to give rise to a population that may help provide a short term bridge for engraftment of an intact unit of cord blood into irradiated patients [6]. However, the detailed nature of those cells is not yet clear. In addition, the Notch-ligand expansion approach only appears to work with cord blood and embryonic liver cells, but not on cells obtained from adult sources [4]. The aryl-hydrocarbon derivatives appear to be very effective in preventing the differentiation of HSCs in culture, but needs another component to help expand the numbers of HSCs *ex vivo*. Lastly, Hox-B4 has been shown to enlarge the number of HSCs in culture [7]. The development of a Tat-fusion protein approach similar to what we describe here has been difficult, as a result of the very short half life of the purified recombinant Tat-HoxB4 protein in culture [7]. The ability of Tat-MYC and Tat-Bcl-2 to increase the total number of HSPCs in culture, from a variety of neonatal and adult sources, while maintaining their multipotential differentiation capacity are key distinguishing features from the previously reported approaches [4]–[5]. Finally, because small amounts of Tat-MYC and Tat-Bcl-2 is required to expand long-term HSCs and Tat-MYC/Tat-Bcl-2 are added directly to the culture media, this approach may be amenable to scale-up in a variety of systems.

The approach described here holds promise for translation to clinical applications for human stem cell transplantation. The main obstacles in current stem cell therapies include the limited cell numbers and identification of allogeneically appropriate donors. The ability to generate large numbers of HSPCs will solve the supply problem. In addition, the ability to transplant a more pure population of stem cells should allow reconstitution of irradiated hosts across allogeneic barriers. Our approach does not require isolation and purification of the CD34^+^ fraction prior to expansion *ex vivo*. This will significantly reduce the cost and simplify applications of this stem cell expansion approach to clinical practice. Further, the potential to apply this expansion strategy to other types of stem cells may help resolve the critical problem of cell number that complicates studies of their biology at a molecular level, as well as enable clinical applications.

## Supporting Information

Figure S1 **Plasmids encoding either Tat-MYC or Tat-Bcl-2 were transduced into E. coli and induced in order to produce recombinant purified proteins.** SDS-PAGE electrophoresis and Coomassie Staining revealed the level of purity of the final product used for our studies.
(TIF)

Figure S2 **C57BL/6J splenic T-cells cultured in the presence of monoclonal antibodies to mouse CD3 were left untreated (NT) or treated with Tat-Cre (TC), Tat-MYC (TM), or Tat-MYC/Tat-Bcl-2 (TMTB).** 48 hrs post transduction, mRNA was isolated and cDNA generated to assess for ODC and GAPDH transcript levels by qPCR.
(TIF)

Figure S3 **Mouse splenic T-cells and B-cells, from a Rag1^−/−^ mouse transplanted with expanded bone marrow cells from 5FU treated C57BL/6J, were labeled with CFSE and cultured in the presence of monoclonal antibodies to mouse CD3 or CD40 and IgM respectively.** Cells were analyzed by FACS 48 hours. Mouse T-cells (first panel black line) and B-cells (second panel black line) that developed in Rag1^−/−^ mice transplanted with expanded BM cells from 5FU treated C57BL/6J underwent proliferation following stimulation of their antigen receptor compared to unstimulated cells (gray line).
(TIF)

Figure S4 **Human splenic B-cells from a NSG mouse, transplanted with expanded cord blood derived HSPCs, were labeled with CFSE and cultured in the presence of monoclonal antibodies to human CD40 and IgM. Cells were analyzed by FACS 72 hours later, showing that human B-cells that developed in NSG xenochimaeric mice underwent proliferation following stimulation of their antigen receptor.**
(TIF)

## References

[1] Spangrude GJ, Heimfeld S, Weissman IL (1988) Purification and characterization of mouse hematopoietic stem cells. Science 241: 58–62. 2898810 10.1126/science.2898810

[2] Morrison SJ, Weissman IL (1994) The long-term repopulating subset of hematopoietic stem cells is deterministic and isolatable by phenotype. Immunity 1: 661–673. 7541305 10.1016/1074-7613(94)90037-x

[3] Copelan EA (2006) Hematopoietic stem cell transplantation. NEJM 354: 1813–1826. 16641398 10.1056/NEJMra052638

[4] Bernstein ID, Delaney C (2012) Engineering stem cell expansion. Cell Stem Cell 10: 113–114. 22305560 10.1016/j.stem.2012.01.012

[5] Chou S, Chu P, Hwang W, Lodish H (2010) Expansion of hematopoietic stem cells for transplantation. Cell Stem Cell 7: 427–428. 20887947 10.1016/j.stem.2010.09.001 PMC2962561

[6] Dahlberg A, Delaney C, Bernstein ID (2011) Ex vivo expansion of human hematopoietic stem and progenitor cells. Blood 117: 6083–6090. 21436068 10.1182/blood-2011-01-283606 PMC3122936

[7] Krosl J, Austin P, Beslu N, Kroon E, Humphries RK, et al (2003) In vitro expansion of hematopoietic stem cells by recombinant Tat-HOXB4 protein. Nat Med 9: 1428–1432. 14578881 10.1038/nm951

[8] Domashenko AD, Danet-Desnoyers G, Aron A, Carroll MP, Emerson SG (2010) Tat-mediated transduction of NF-Ya peptide induces the ex vivo proliferation and engraftment potential of human hematopoietic progenitor cells. Blood 116: 2676–2683. 20616221 10.1182/blood-2010-03-273441 PMC2974580

[9] Yang J, Aguila JR, ALipio Z, Lai R, Fink LM, et al (2011) Enhanced self-renewal of hematopoietic stem/progenitor cells mediated by the stem cell gene Sall4. J Hematol Oncol 4: 38. 21943195 10.1186/1756-8722-4-38 PMC3184628

[10] North TE, Goessling W, Walkley CR, Lengerke C, Kopani KR, et al (2007) Prostaglanding E2 regulates vertebrate haematopoietic stem cell homeostasis. Nature 447: 1007–1011. 17581586 10.1038/nature05883 PMC2775137

[11] Boitano AE, Wang J, Romeo R, Bouchez LC, Parker AE, et al (2010) Aryl hydrocarbon receptor antagonists promote the expansion of human hematopoietic stem cells. Science 329: 1345–1348. 20688981 10.1126/science.1191536 PMC3033342

[12] Walasek MA, van Os R, de Haan G (2012) Hematopoietic stem cell expansion: challenges and opportunities. Ann NY Acad Sci 1266: 138–150. 22901265 10.1111/j.1749-6632.2012.06549.x

[13] Turner BC, Eves T, Refaeli Y (2008) Small-molecule inhibitors of Bcl-2 family proteins are able to induce tumor regression in a mouse model of pre-B-cell acute lymphocytic lymphoma. DNA Cell Biol 3: 133–142. 10.1089/dna.2007.0675 18163880

[14] Refaeli Y, Young RM, Turner BC, Duda J, Field KA, et al (2008) The B-cell antigen receptor and overexpression of MYC can cooperate in the genesis of B-cell lymphomas. PLoS Biology 6: e152. 18578569 10.1371/journal.pbio.0060152 PMC2435152

[15] Young RM, Turner BC, Refaeli Y (2008) B-cell receptor signaling in the genesis and maintenance of B-cell lymphoma. Future Oncology 4: 591–594. 18922114 10.2217/14796694.4.5.591 PMC2575750

[16] Anderson M, Blowers D, Hewitt N, Hedge P, Breeze A, et al (1999) Refolding, purification and characterization of a loop deletion mutant of human Bcl-2 from bacterial inclusion bodies. Prot Expr. Purif 15: 162–170. 10.1006/prep.1998.0996 10049671

[17] Bello-Fernandez C, Packham G, Cleveland JL (1993) The ornithine decarboxylase gene is a transcriptional target of c-Myc. Proc Natl Acad Sci USA 90: 7804–7808. 8356088 10.1073/pnas.90.16.7804 PMC47231

[18] Refaeli Y, Field KA, Turner BC, Trumpp A, Bishop JM (2005) The proto-oncogene MYC can break B-cell tolerance, Proc Natl Acad Sci USA. 102: 4097–4102. 10.1073/pnas.0409832102 PMC552974 15753301

[19] Lerner C, Harrison DE (1990) 5-Fluorouracil spares hemopoietic stem cells responsible for long-term repopulation. Exp Hematol 18: 114–118. 2303103

[20] Yeager AM, Levin J, Levin FC (1983) The effects of 5-fluorouracil on hematopoiesis: study of murine megakaryocyte-CFC, granulocyte-macrophage-CFC, and peripheral blood cell levels. Exp Hematol 11: 944–952. 6662215

[21] Vetvicka V, Kincade PW, Witte PL (1986) Effects of 5-fluorouracil on B lymphocyte lineage cells. J Immunol 137: 2405–2410. 3093575

[22] Suzuki T, Yokoyama Y, Kumano K, Takanashi M, Kozuma S, et al (2006) Highly efficient ex vivo expansion of human hematopoietic stem cells using delta-fc chimeric protein. Stem Cells 24: 2456–2465. 16857897 10.1634/stemcells.2006-0258

[23] Walasek MA, vanOs R, de Haan G (2012) Hematopoietic stem cell expansion: challenges and opportunities. Ann N Y Acad Sci 1266: 138–150. 22901265 10.1111/j.1749-6632.2012.06549.x

[24] Sideri A, Neokleous N, Brunet De La Grange P, Guerton B, Le Bousse Kerdilles MC, et al (2011) An overview of the progress on double umbilical cord blood transplantation. Hematologica 96: 1213–1220. 10.3324/haematol.2010.038836 PMC3148916 21546497

