# Supplementary figures and images for: Expansion of Human and Murine Hematopoietic Stem and Progenitor Cells Ex Vivo without Genetic Modification Using MYC and Bcl-2 Fusion Proteins
Source: PLoS One. 2014 Aug 29;9(8):e105525. doi: 10.1371/journal.pone.0105525 (PMC4149411; doi:10.1371/journal.pone.0105525)

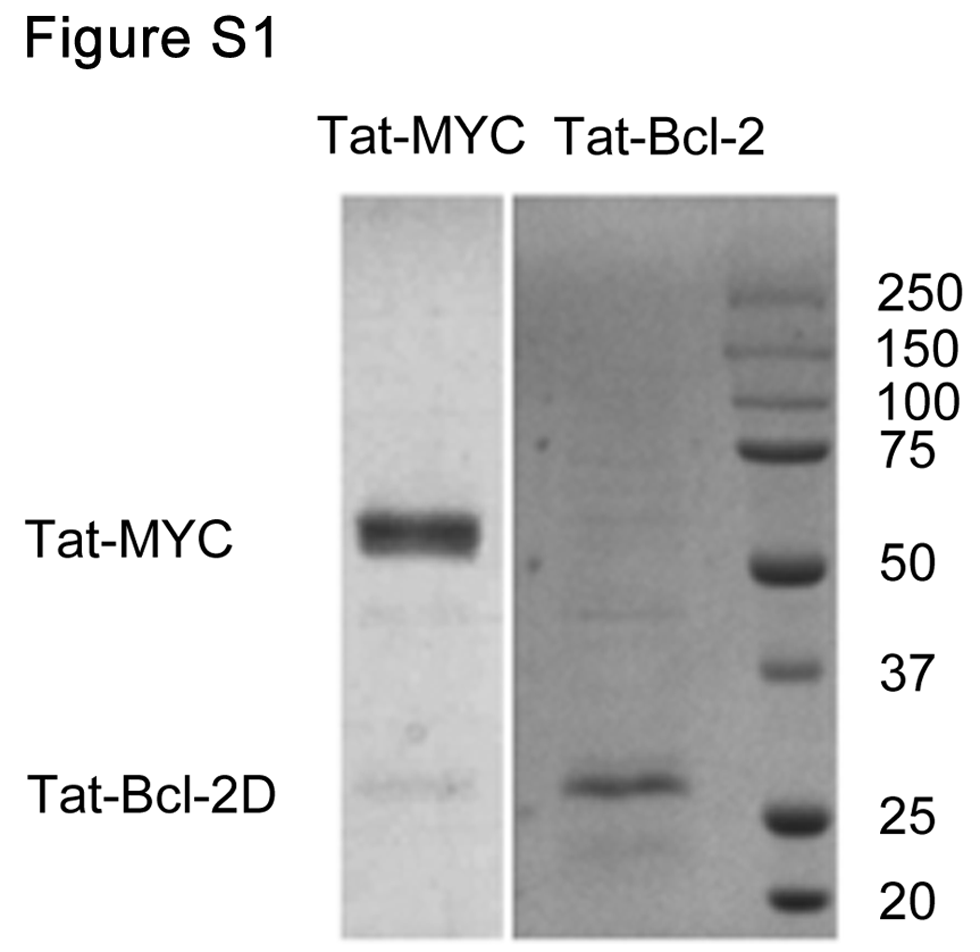

Supplement: Figure S1 — Plasmids encoding either Tat-MYC or Tat-Bcl-2 were transduced into E. coli and induced in order to produce recombinant purified proteins. SDS-PAGE electrophoresis and Coomassie Staining revealed the level of purity of the final product used for our studies. (TIF) [file pone.0105525.s001.tif]

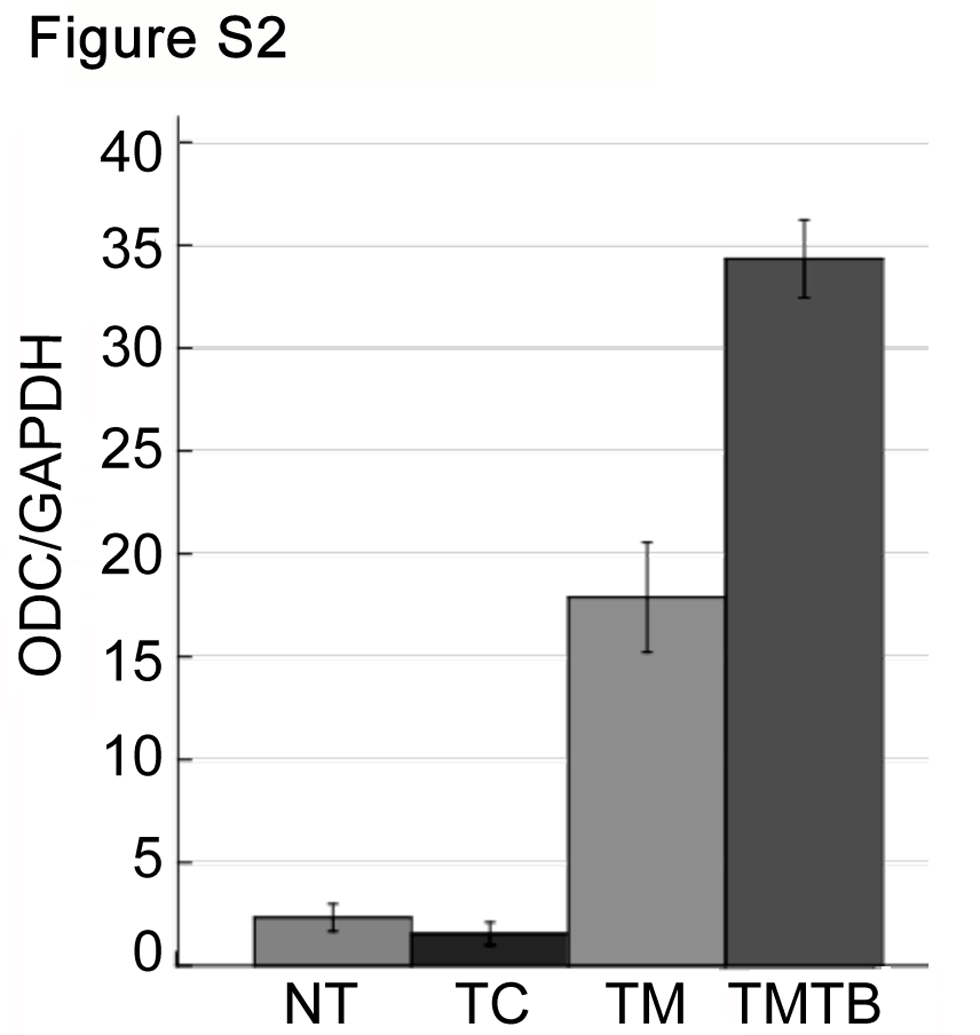

Supplement: Figure S2 — C57BL/6J splenic T-cells cultured in the presence of monoclonal antibodies to mouse CD3 were left untreated (NT) or treated with Tat-Cre (TC), Tat-MYC (TM), or Tat-MYC/Tat-Bcl-2 (TMTB). 48 hrs post transduction, mRNA was isolated and cDNA generated to assess for ODC and GAPDH transcript levels by qPCR. (TIF) [file pone.0105525.s002.tif]

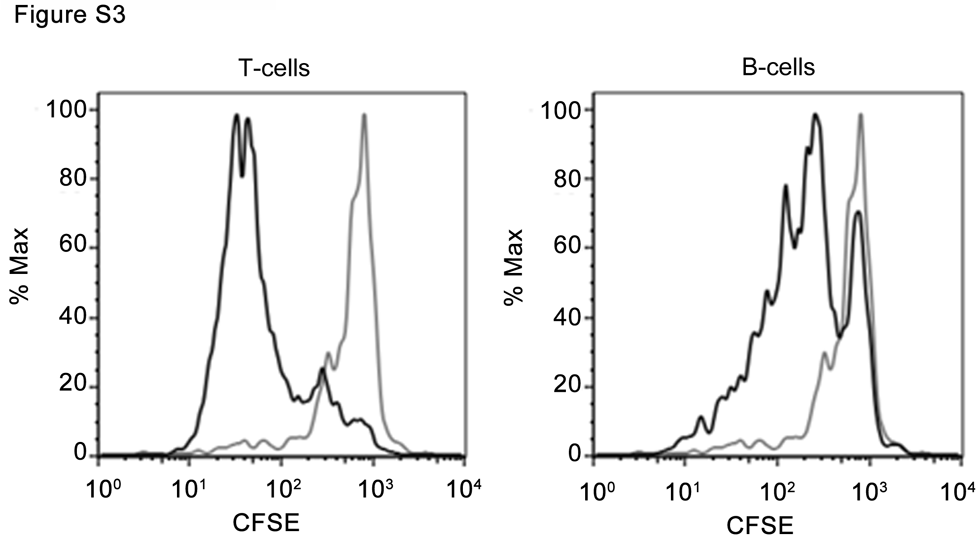

Supplement: Figure S3 — Mouse splenic T-cells and B-cells, from a Rag1−/− mouse transplanted with expanded bone marrow cells from 5FU treated C57BL/6J, were labeled with CFSE and cultured in the presence of monoclonal antibodies to mouse CD3 or CD40 and IgM respectively. Cells were analyzed by FACS 48 hours. Mouse T-cells (first panel black line) and B-cells (second panel black line) that developed in Rag1−/− mice transplanted with expanded BM cells from 5FU treated C57BL/6J underwent proliferation following stimulation of their antigen receptor compared to unstimulated cells (gray line). (TIF) [file pone.0105525.s003.tif]

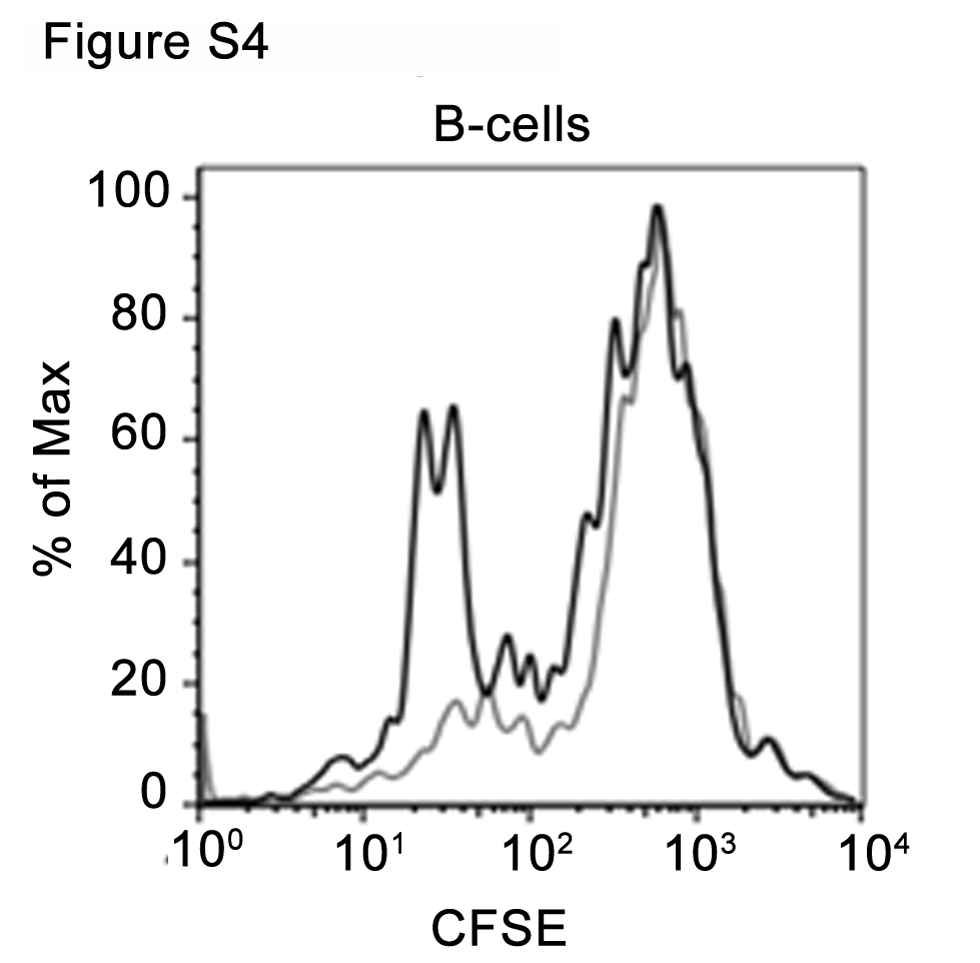

Supplement: Figure S4 — Human splenic B-cells from a NSG mouse, transplanted with expanded cord blood derived HSPCs, were labeled with CFSE and cultured in the presence of monoclonal antibodies to human CD40 and IgM. Cells were analyzed by FACS 72 hours later, showing that human B-cells that developed in NSG xenochimaeric mice underwent proliferation following stimulation of their antigen receptor. (TIF) [file pone.0105525.s004.tif]
